# A Janus Smart Window for Temperature-Adaptive Radiative Cooling and Adjustable Solar Transmittance

**DOI:** 10.1007/s40820-025-01740-1

**Published:** 2025-04-27

**Authors:** Zuowei Zhang, Meina Yu, Cong Ma, Longxiang He, Xian He, Baohua Yuan, Luoning Zhang, Cheng Zou, Yanzi Gao, Huai Yang

**Affiliations:** 1 Institute for Advanced Materials and Technology, University of Science and Technology, Beijing, 100083 People’s Republic of China; 2 Beijing Advanced Innovation Center for Materials Genome Engineering, University of Science and Technology, Beijing, 100083 People’s Republic of China; 3 School of Materials Science and Engineering, Peking University, Beijing, 100871 People’s Republic of China

**Keywords:** Thermal insulation, Solar modulation, Photothermal conversion, Radiative cooling, Energy saving

## Abstract

The advancement of sophisticated smart windows exhibiting superior thermoregulation capabilities in both solar spectrum and long-wave infrared range maintains a prominent objective for researchers in this field. In this study, a Janus window is proposed and prepared based on polymer-stabilized liquid–crystal films/thermochromic materials. It can achieve switchable front long-wave infrared emissivity (*ε*_Front_) and solar modulation ability (Δ*T*_sol_) through dynamic flipping, making it suitable for different seasonal energy-saving requirements. Outdoor experiments show that under daytime illumination, the indoor temperature decreases by 8 °C, and the nighttime temperature drops by 5 °C. MATLAB simulation calculations indicate that the daytime cooling power is 93 W m^−2^, while the nighttime cooling power reaches 142 W m^−2^. Interestingly, by modifying the conductive layer, it can effectively shield electromagnetic radiation (within the X-band frequency range (8.2–12.4) GHz). Energy simulation reveals the substantial superiority of this device in energy savings compared with single-layer polymer-stabilized liquid crystal, poly(N-isopropyl acrylamide), and normal glass when applied in different climate zones. This research presents a compelling opportunity for the development of sophisticated smart windows characterized by exceptional thermoregulation capabilities.

**Supplementary Information:**

The online version contains supplementary material available at 10.1007/s40820-025-01740-1.

## Highlights

- Using polymer-stabilized liquid crystal as the active control layer and KCWO-PNIPAM (KPP) as the passive control layer, a KPP smart window was constructed, successfully achieving dual electro-thermal collaborative control.
- The window can adjust incident light across three bands: visible light, near-infrared light, and mid-far infrared light (especially 8–13 µm).
- Combination of active and passive control to meet the diverse weather conditions.

**Supplementary Information:**

The online version contains supplementary material available at 10.1007/s40820-025-01740-1.

## Graphical abstract

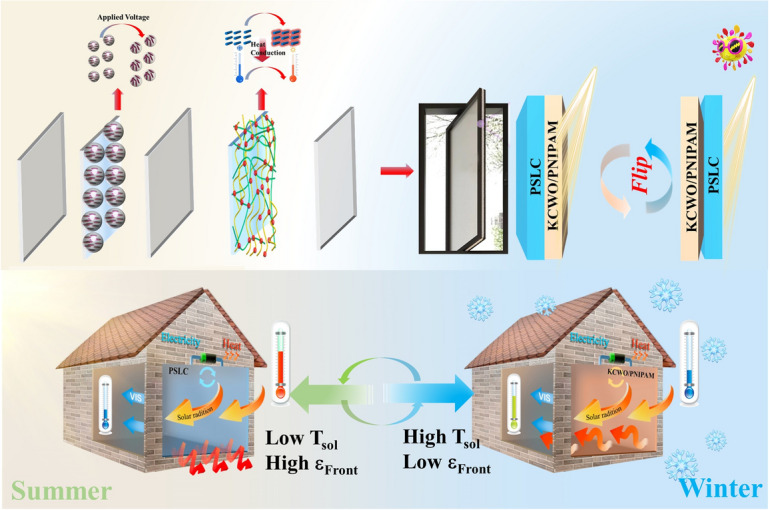

## Introduction

**Fig. 1:**
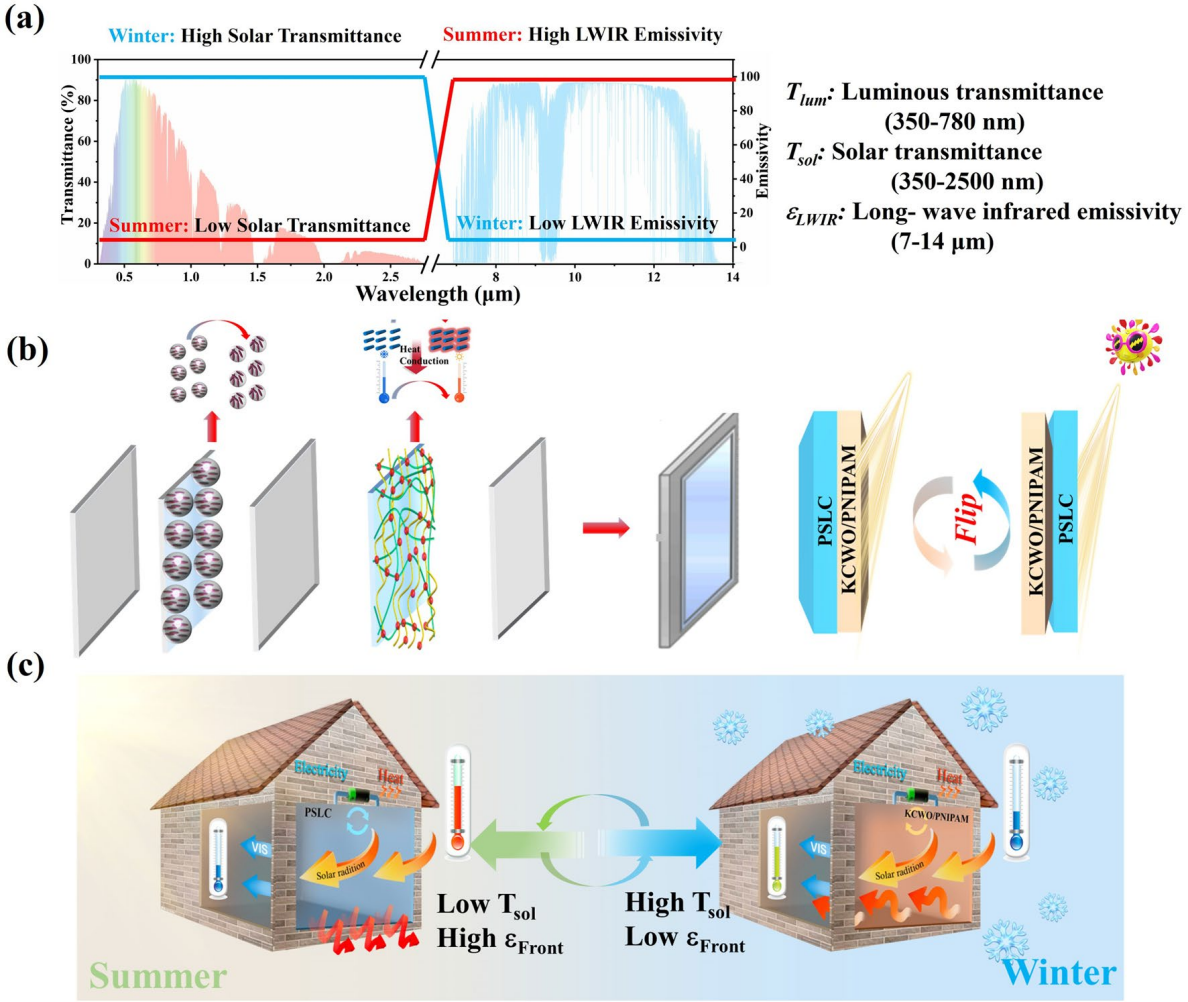
**a** Concept of the ideal smart window in energy-saving mode for summer (red line in spectra) and winter (blue line in spectra) for wavelength ranges of UV, vis, NIR and LWIR; and important parameters: luminous transmittance (*T*_lum_), solar transmittance (*T*_sol_), and LWIR emissivity (*ε*_LWIR_) for the energy-saving smart window; **b** the schematic illustration for the fabrication and modulation mechanism of smart windows; **c** schematic illustration for the fabrication and modulation mechanism of smart windows

Approximately 40% of the world's energy consumption is attributed to energy use in buildings and the main culprits are heating, ventilation and air-conditioning (HVAC) [1, 2]. The research report of China building energy consumption and carbon emissions highlighted that carbon dioxide emissions from buildings reached 22.22 tons in 2022, representing approximately 20% of the country’s total carbon emissions. Among these, energy losses through glazing, windows, and doors account for 5% of the total building energy consumption. In today’s “carbon–neutral” context, the trend of green and energy-efficient buildings has become a necessity for environmental protection and market development. Windows play a crucial role as the primary channel for heat exchange between the indoor environment and the outside world in green buildings. They are the primary light-gathering components of a building, but they are also the weakest link in the thermal insulation of the building envelope, resulting in a low energy utilization rate [3–5]. In winter, the heat lost through glass windows accounts for 30%–50% of the heating load, and in summer, the refrigeration consumption caused by the passage of radiative heat from sunlight through glass windows accounts for 20%–30% of the air-conditioning load [6, 7]. Hence, it is crucial to minimize heat loss from windows and enhance the efficiency of solar energy utilization. Smart windows, which can adjust the amount of solar radiation passively or actively, showcase significant potential for application in green buildings. A perfect smart window should be able to control three wave bands of sunlight, namely visible (0.38–0.78 μm), near-infrared (NIR, 0.78–2.5 μm), and the long-wave infrared (LWIR, 2.5–25 µm) bands. Visible and NIR transmittance (0.38–2.5 µm) determines the indoor solar heat gain, whereas LWIR thermal emittance (*ε*_LWIR_) dominates the radiative cooling (RC) to the outer spaces. An ideal energy-saving smart window should have a low solar transmittance (*T*_sol_) and a high *ε*_LWIR_ in summer to minimize the heat gain of the building through the window, as shown in Fig. 1a. Conversely, low *ε*_LWIR_ and high *T*_sol_ are beneficial to provide heat gain and suppress RC in winter.

Essentially, these technologies are primarily categorized into electrochromic [8–12], photochromic [6, 13, 14], thermochromic [15–18], and liquid crystal (LC) types [19–24]. Presently, there is a significant emphasis on thermochromic materials such as VO_2_, poly(N-vinylcaprolactam), poly(methyl vinyl ether), chitosan, hydroxypropylllcellulose, polythiophene, poly(N-isopropyl acrylamide) (PNIPAM), and poly (N-vinyl-caprolactam) [25–31]. Among them, PNIPAM is commonly used because of its lower critical solution temperature (LCST) of 32 °C. This temperature range aligns well with the typical building temperatures of 25–35 °C, making PNIPAM suitable for use in various regions with hot summer climates [32–35]. Zhouet al. have developed a new type of window by injecting a hydrogel between two glass panels, which exhibits excellent thermoresponsive optical properties [36]. Zhang et al. successfully doped V_0.8_W_0.2_O_2_@SiO_2_ into PNIPAM [37]. When the inclusion content was 1 wt%, the modulation capability to modulate sunlight reached 76.8%. By applying a cesium tungstate thin film to the surface of hydrogel, Wu et al. successfully obtained a kind of intelligent window with dual-band regulation that exhibits an excellent near-infrared shielding effect [38]. Although the PNIPAM smart window has shown promising solar modulation ability (Δ*T*_sol_), it is still far from the ideal smart window as the design of conventional chromogenic smart window does not consider the effect of regulating front LWIR emissivity (*ε*_Front_) that represents the ε_LWIR_ of the outer side of the window.

Radiative cooling is a technique that spontaneously cools a surface by radiating LWIR to the cold outer space. This can be achieved through high ε_LWIR_ materials [39, 40]. Previous reports have also proposed many related to the preparation and application of radiative refrigeration materials, this significantly reduces the use of appliances such as air-conditioning, leading to a substantial decrease in energy consumption [41–45]. Xiang and colleagues prepared a fiber-reinforced composite film. The resulting membrane presents outstanding outdoor thermoregulation capacity, with a practically attainable sun-ambient cooling temperature of ~ 4.5 °C [46]. Zhang and colleagues successfully developed a textile using electrospinning and spraying techniques, which exhibited superior cooling effects in outdoor environments compared to commercial cotton [47]. For areas with four distinct seasons, winter heating and summer cooling are equally important. Thus, the ε_LWIR_ of the material must be adjusted. Besides, the thermochromic smart window is passively regulated and relies heavily on weather conditions. Polymer-stabilized liquid crystal (PSLC), as a type of LC-based smart window, can achieve active regulation. The film exhibits a transparent appearance under zero-field conditions. When exposed to an external voltage, the LCs are rotated by the electric field, and an opaque state is displayed. At the same time, the presence of a large number of C–F, C–H, and C–O bonds in LCs and polymers endows the films with a high emissivity in the LWIR range, enabling radiation cooling effects [48, 49]. A limitation of this material is that its *ε*_LWIR_ is constant and cannot be modified, thereby constraining its applicability in winter and other cold climates.

In addition to regulating visible light, NIR light, and mid-to-far infrared wavelengths, the ability to control electromagnetic waves is crucial for the development of future smart windows [50]. The 5G mobile network offers endless possibilities for low-latency and high-speed applications. However, the expansion of frequency ranges, increased energy levels, and higher electromagnetic energy density raise concerns about public health and the stability of electronic systems. Wang and colleagues reported a visible-infrared-radar multi-band camouflage MXene nanocomposite cholesteric liquid crystal smart material inspired by cephalopods. The synthesized MXene-CLCE demonstrates dynamic structural color changes, adjustable infrared radiation, and switchable microwave shielding properties. It has demonstrated significant potential in areas such as military stealth and dynamic thermal management [51]. Currently, the capability for electromagnetic shielding in the field of smart windows has been largely overlooked [52]. Therefore, there is an urgent need for windows that can simultaneously provide solar modulation, heat regulation, and electromagnetic shielding functions.

In this study, we demonstrate a practical co-assembly approach for producing smart windows, named KCWO-PNIPAM/PSLC smart window (KPP smart window). The solar control functionality is enhanced by fine-tuning the performance of individual components and assembly configuration. In detail, electrochromic PSLC layer is used as the main control switch, while the thermochromic KCWO-PNIPAM (K_0.1_Cs_0.22_WO_3_/PNIPAM) hydrogel layer serves as an auxiliary functional module to achieve a combination of active and passive regulation, as shown in Fig. 1b. The basic concept is illustrated in Fig. 1b, c (the scheme of the structure for Janus smart window panel is shown in Fig. S1). In cold winters, the KPP smart window with KCWO-PNIPAM layer facing the outside exhibits a low *ε*_Front_, which possesses weak radiative cooling effect to ensure indoor temperature stability. At night, the high specific heat capacity of the hydrogel demonstrates outstanding insulation ability to prevent indoor heat loss. In summer, the system can switch to a KPP window with PSLC layer facing the outside. The high *ε*_Front_ increases the radiative cooling capability of the PSLC layer, while the KCWO-PNIPAM layer can absorb infrared light, thereby lowering the indoor temperature. The proposed smart window offers an effective solution to enhance the energy efficiency of buildings throughout both summer and winter. It is particularly beneficial in areas with significant annual temperature fluctuations and can facilitate more adaptable environmental responses. Moreover, the electromagnetic shielding performance of the smart window is validated, and by adjusting the conductive coating, the electromagnetic shielding effect of the smart window can be improved. This provides valuable insights for the production of multifunctional smart windows.

## Concept and Structure

Figure S1 shows the structure of the smart window. The components are mainly consisted of two parts, electrochromic PSLC layer with high *ε*_LWIR_ (active regulation) and thermochromic KCWO-PNIPAM layer with low *ε*_LWIR_ (passive regulation). The electrochromic part consists of substrates (poly(ethylene terephthalate), PET) layer with inner surface coated by indium tin oxide electrode) and PSLC layer. Because of its special network structure and rich chemical bonds, PSLC exhibits high ε_LWIR_. Meanwhile, it can achieve multi-level adjustment of sunlight transmittance by adjusting the voltage to ensure privacy, which is impossible for thermochromic materials. The thermochromic part consists of the KCWO-PNIPAM and low ε_LWIR_ layer (the indium tin oxide thin film deposited on PE), where the low ε_LWIR_ layer was employed to protect the KCWO-PNIPAM from drying because of its good chemical stability. KCWO-PNIPAM is highly transparent at temperatures below the phase transition temperature. As a photothermal converter, KCWO absorbs near-infrared sunlight to achieve efficient photothermal conversion. Once the temperature exceeds its transition temperature, KCWO-PNIPAM hydrogel becomes opaque and blocks sunlight, showing good sunlight regulation capabilities. The KPP smart window possesses dual functionalities of active and passive sunlight regulation and presents different ε_LWIR_ values on two sides. The working principle is illustrated in Fig. 1b, c. The smart window is designed with the flip mode. In hot summer, the high *ε*_Front_ PSLC window faces outward to achieve all-day radiative cooling (red arrow). The sunlight transmittance can be controlled by both electric field and heat. In the morning and evening, the hydrogel is highly transparent. The PSLC layer actively regulates sunlight transmittance to protect indoor privacy. When the temperature is too high at noon, the solar radiation intensity is highest and the hydrogel layer becomes highly opaque, preventing sunlight entry. In winter, the low *ε*_Front_ layer is turned outward to eliminate the cooling of radiation. Because the temperature was too low, the hydrogel cannot change into an opaque state to adjust the solar transmittance. The PSLC layer can be adjusted by voltage to achieve multi-level regulation of sunlight, ensuring uninterrupted indoor privacy at any time. More interestingly, the large specific heat capacity enables the KCWO-PNIPAM layer to exhibit excellent heat storage and insulation, effectively preventing indoor heat loss.

## Results and Discussion

### Characterization and Analysis of the KCWO Nanoparticles

**Fig. 2:**
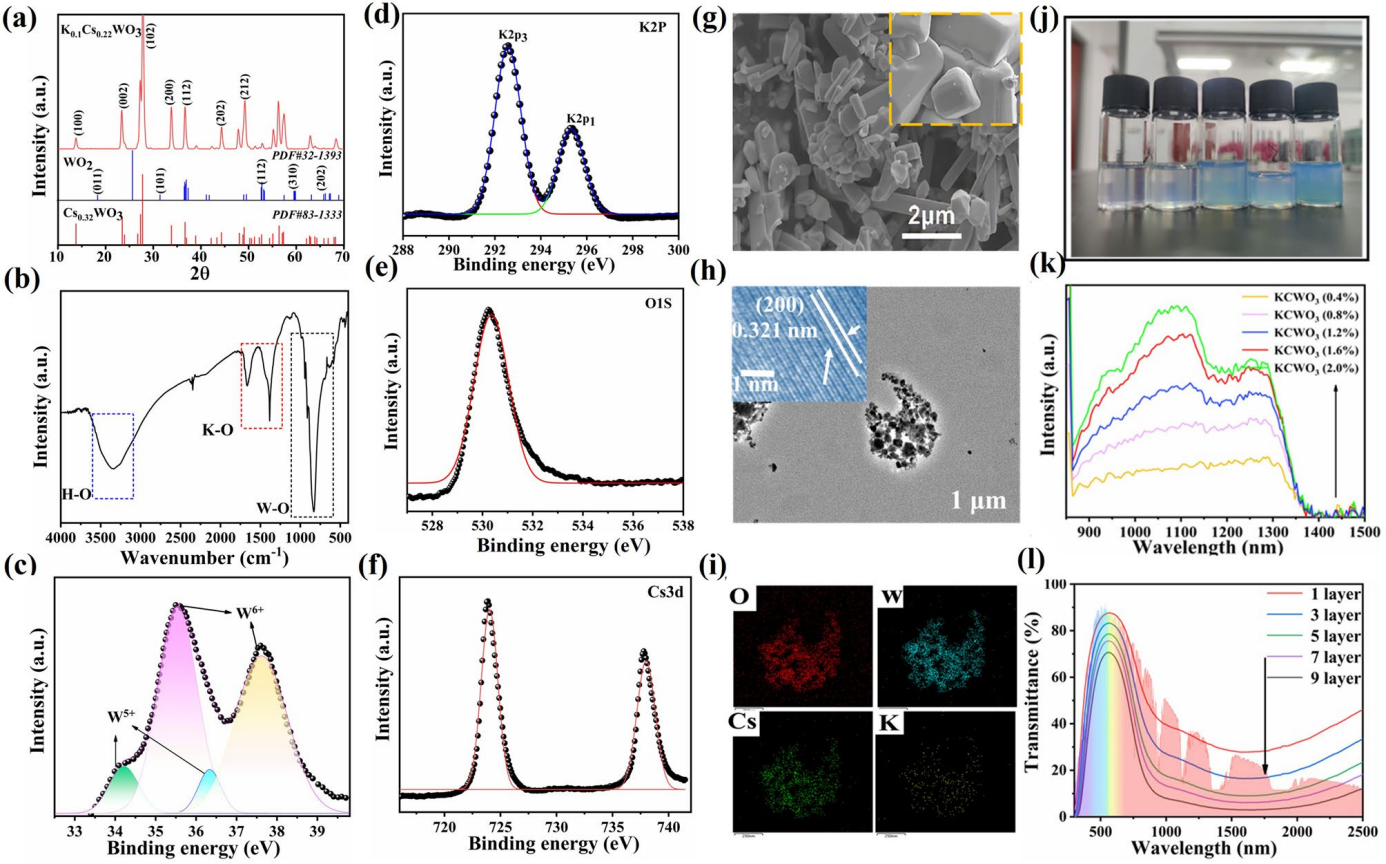
**a** XRD patterns; **b** FTIR spectra of KCWO; **c** XPS of W 4*f* spectrum; XPS of **d** W K 2*p*, **e** O 1*s* and **f** Cs 3*d* spectrum; **g** SEM images of KCWO; **h** TEM images of KCWO; the inset illustration shows the corresponding HRTEM image; **i** EDS elemental analysis; **j** photographs of solutions containing varying concentrations of KCWO particles: 0.4, 0.8, 1.2, 1.6, and 2.0 wt% (left to right); **k** absorption spectrum of different concentrations of KCWO; **l** ultraviolet-visible-near-infrared transmission spectra of glass sample coated with KCWO films

Figure 2a displays the X-ray diffraction (XRD) patterns of the potassium copper tungsten oxide (KCWO) sample. The diffraction peak position of K_m_Cs_n_WO_3_ remains consistent with the Cs_0.32_WO_3_ particle. Meanwhile, additional peaks were identified, corresponding to the (112), (310), and (202) crystal planes of WO_2_, as indicated by PDF#32-1393, which may be attributed to WO_2_ particles produced by hydrogen reduction. In order to demonstrate the successful synthesis of KCWO nanoparticles, the FTIR spectra were measured are and displayed in Fig. 2b. The observed peak at 3478 cm^−1^ is assigned to the vibrational mode of the hydroxyl group (O–H). The peaks at 1450 and 1520 cm^−1^ are associated with the K–O characteristic, while the peak at 810 cm^−1^ is due to the W–O characteristic [53, 54]. The surface elements of the as-synthesized sample were investigated by XPS analysis. In Fig. 2d, the peaks K-2P1 at 292.3 eV and K-2P3 at 295.2 eV may be due to the oxidation state of K. The binding energy of O 1*s* is 531.21 eV in Fig. 2e. The peaks Cs-3*d*_3/2_ at 724.13 eV and Cs-3*d*_5/2_ at 738.12 eV could be attributed to the oxidation state of Cs as shown in Fig. 2f. It is worth noting that the W-4*f* core-level spectrum is presented in Fig. 2c, which can be fitted as two spin–orbit doublets with a separation interval of 2 eV. The binding energy peaks at 37.1 and 35.1 eV can be assigned to W^6+^, while the peaks at 36.4 and 34.4 eV are caused by the presence of W^5+^ [55, 56]. After calculations, it was determined that the ratio of W^5+^ to W^6+^ is 0.13–1. The SEM images of the KCWO sample are shown in Fig. 2g. The rod-shaped KCWO will facilitate the higher near-infrared absorption. At higher magnification, as shown in the inset illustration, the reduction in particle size of KCWO particles was notable due to the ball milling process, while the lattice structure of the particles remained unchanged. The lattice spacing of 0.321 nm corresponds to the (200) plane of KCWO, as shown in Fig. 2h. The energy-dispersive X-ray spectroscopy (EDS) is observed in Fig. 2f, corresponding to the area in Fig. 2i. The successful synthesis of KCWO is confirmed by the detection of potassium (K), oxygen (O), tungsten (W), and cesium (Cs) elements in the sample. In Fig. 2j, the images illustrate the dispersion of KCWO in a PVA solution at different mass fractions of 0.4, 0.8, 1.2, 1.6, and 2.0 wt%, respectively. Figure 2k depicts the absorption peak associated with the aforementioned samples. The study demonstrates that the peak value in the NIR spectrum increases with the rise in the mass fraction of KCWO. Figure 2l illustrates the ultraviolet-visible-near-infrared transmission spectra of KCWO films that were coated on glass substrates. It is observed that with an increase in the film thickness, there was a progressive enhancement in the near-infrared shielding capability. The results indicate that the synthesized KCWO particles exhibit a high capacity for infrared absorption.

### Passive Regulation of KPP Smart Window-KCWO/PNIPAM Hydrogel Layer

**Fig. 3:**
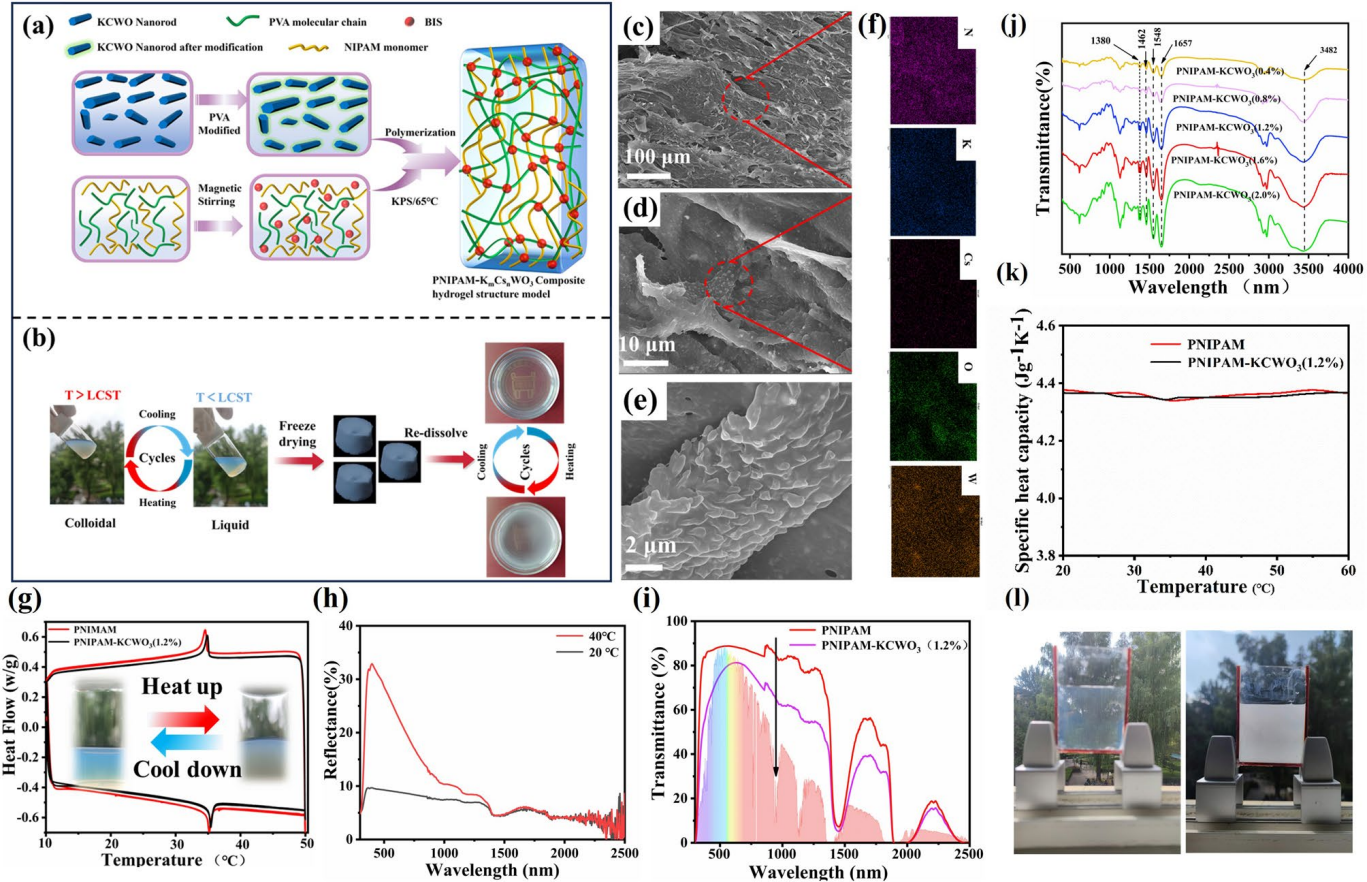
**a** Formation mechanism of hydrogel, **b** illustration depicting the phase transformation process of a hydrogel. **c** SEM images of KCWO/PNIPAM (1.2%); **d** enlarged SEM image corresponding to the red area in **c**; **e** enlarged SEM image corresponding to the red area in **c**; **f** EDS spectra of KCWO/PNIPAM **c**; **g** DSC spectra of KCWO composite hydrogels by the way of freeze-drying under controlling the different contents of KCWO from 0.4 to 2.0 wt%; **h** reflectance of KCWO/PNIPAM (1.2%) at full solar spectrum at 20 and 40 °C; **i** transmittance of PNIPAM and KCWO/PNIPAM (1.2%) at full solar spectrum at 20 and 40 °C; **j** FTIR spectra of PNIPAM, and KCWO/PNIPAM; **k** the value of specific heat capacity of KCWO/PNIPAM and PNIPAM; **l** photographs of PNIPAM/KCWO layer

Figure 3a illustrates the preparation process and structure of KCWO/PNIPAM hydrogels. Firstly, the KCWO nanoparticles were dispersed into 5 wt% PVA solution to ensure uniform dispersion. Then, the NIPAM and bisphenol A ethoxylate (BIS) monomers were added and thoroughly mixed through blending, and finally the KCWO/PNIPAM hydrogel was obtained after the completion of the reaction. As shown in Fig. 3a, the interpenetrating network structure is created by the entanglement of PVA molecular chains and NIPAM monomers, leading to a significant improvement in the stability of the hydrogel. Meanwhile, KCWO nanoparticles are coated with polyvinyl alcohol (PVA) and hydrogel networks, which effectively prevent them from precipitating after prolonged use, thereby enhancing their dispersion and stability within the hydrogel (we compared the dispersibility of KCWO nanoparticles with PVA-modified KCWO in aqueous hydrogels, as shown in supporting information). Hydrogel molecules contain a large number of amino groups on their side chains, which can form hydrogen bonds with water molecules and are hydrophilic at low temperatures. At high temperatures, the methyl and amino groups on the side chains of the hydrogel molecules shrink inward, reducing the opportunity to form hydrogen bonds with water molecules. Consequently, the hydrogel becomes hydrophobic and appears opaque. To enable the observation of hydrogels’ microscopic morphology and their storage and transportation in practical applications, solid powdery hydrogels were produced by freeze-drying process. As depicted in Fig. 3b, the freeze-dried hydrogel re-formed a uniform KCWO/PNIPAM composite hydrogel after being dissolved in water again. The redissolved KCWO/PNIPAM composite hydrogels maintain excellent thermal responsiveness and phase transition, implying good stability. Figure 3c–e displays the SEM microstructure of the KCWO/PNIPAM hydrogel. It can be found that the as-prepared KCWO/PNIPAM hydrogel mainly exhibits an irregular flake appearance. As shown in Fig. 3d, e, the hydrogels exhibit a significant number of small protrusions on their surface. Subsequently, the bump was analyzed by EDS energy spectroscopy, as shown in Fig. 3f. The analysis revealed that the protrusion predominantly consists of potassium (K), cesium (Cs), tungsten (W), oxygen (O), and nitrogen (N) elements. Additionally, the uniform distribution of elements K, Cs, W, and O indicates that the KCWO particles are uniformly distributed within the hydrogel matrix. Figure 3g shows the differential scanning calorimetry (DSC) test results of PNIPAM and KCWO/PNIPAM hydrogels. Slight differences can be observed between the two samples, suggesting that nanoparticles exhibit minimal influence on the phase transition temperature of hydrogel. The LCST is 35.12 °C for the KCWO/PNIPAM sample and 34.96 °C for the pristine PNIPAM sample. The temperature is very close to the human body’s comfort level and is ideal for use as the switching temperature for smart windows. Figure 3h shows the reflectance of KCWO/PNIPAM (1.2 wt%) at full solar spectrum at 20 and 40 °C. The ultraviolet (UV)-visible (Vis)-near-infrared (NIR) transmission spectra of the KCWO/PNIPAM composite hydrogel before the phase transition are shown in Fig. 3i. It can be observed that compared with pure PNIPAM, the transmittance of KCKWO/PNIPAM at 1000–2500 nm band is significantly reduced, indicating that KCWO exhibits excellent near-infrared absorption ability (the transmittance and reflectance of PNIPAM at full solar spectrum with different mass fractions of KCWO at 25 and 40 °C are shown in Fig. S4). It can be observed in Fig. 3j that the FTIR spectra of various samples are nearly identical, indicating that the inclusion of KCWO nanoparticles did not impact the structure of the PNIPAM hydrogel. The peaks at 3482 and 3270 cm^−1^ could be assigned to the vibration of the O–H bond and the N–H stretching vibration [57], respectively. The peaks observed at 1380 and 1462 cm^−1^ are associated with the vibrational modes of the C–H bond within the isopropyl group. Furthermore, the peak value detected at 1548 and 1657 cm^−1^ are associated with amide II (N–H) group of PNIPAM and the amide I bond (C = O), respectively. The aforementioned findings suggest that the KCWO/PNIPAM hydrogel was effectively synthesized [58]. Figure 3k displays the specific heat capacity of the KCWO/PNIPAM, showcasing its exceptional heat storage capability. This is essential for the practical implementation of smart window technology. The photographs of the PNIPAM/KCWO layer are shown in Fig. 3l.

The response time of the KCWO/PNIPAM hydrogel is studied, as shown in Fig. S5a. During the test, the environmental temperature was recorded as 25 °C. The infrared light source was positioned at a distance of 10 cm from the smart window, with emitting intensities of 0.637 and 1.467 mW cm^−2^, respectively. After 8 min of irradiation, the smart window switches completely from the on state to the off state at a light intensity of 1.467 mW cm^−2^. Conversely, only a fraction of the composite hydrogel underwent a phase shift at a lower light intensity of 0.637 mW cm^−2^. Figure S4b illustrates the UV–Vis-NIR transmission spectra at different temperatures. When the temperature reaches 35 °C, the smart window switches to an opaque state. The above results indicate that thermochromic layers exhibit rapid response and hold significant potential for enhancing energy efficiency and reducing emissions in contemporary applications. Furthermore, the durability was evaluated. To assess the stability of the sample during practical application, we performed 150 cycles of high-temperature phase transition testing, ranging from 25 to 55 °C, and 150 cycles of low-temperature testing, spanning from 25 to  − 10 °C. The results of the cycle tests, which measured transmittance at 550 and 1400 nm for the composite hydrogel smart window under both high and low-temperature conditions, are presented in Fig. S4c, d, respectively. The findings indicate that temperature variations have a minimal impact on the near-infrared (NIR) transmission and shielding capabilities of the composite hydrogel smart window. The insets of Fig. S4c, d display actual photographs of the composite hydrogel sandwich glass at  − 10, 25, and 55 °C post-durability testing, further confirming the hydrogel’s remarkable stability.

### Active Regulation of KPP Smart Window-PSLC Layer

**Fig. 4:**
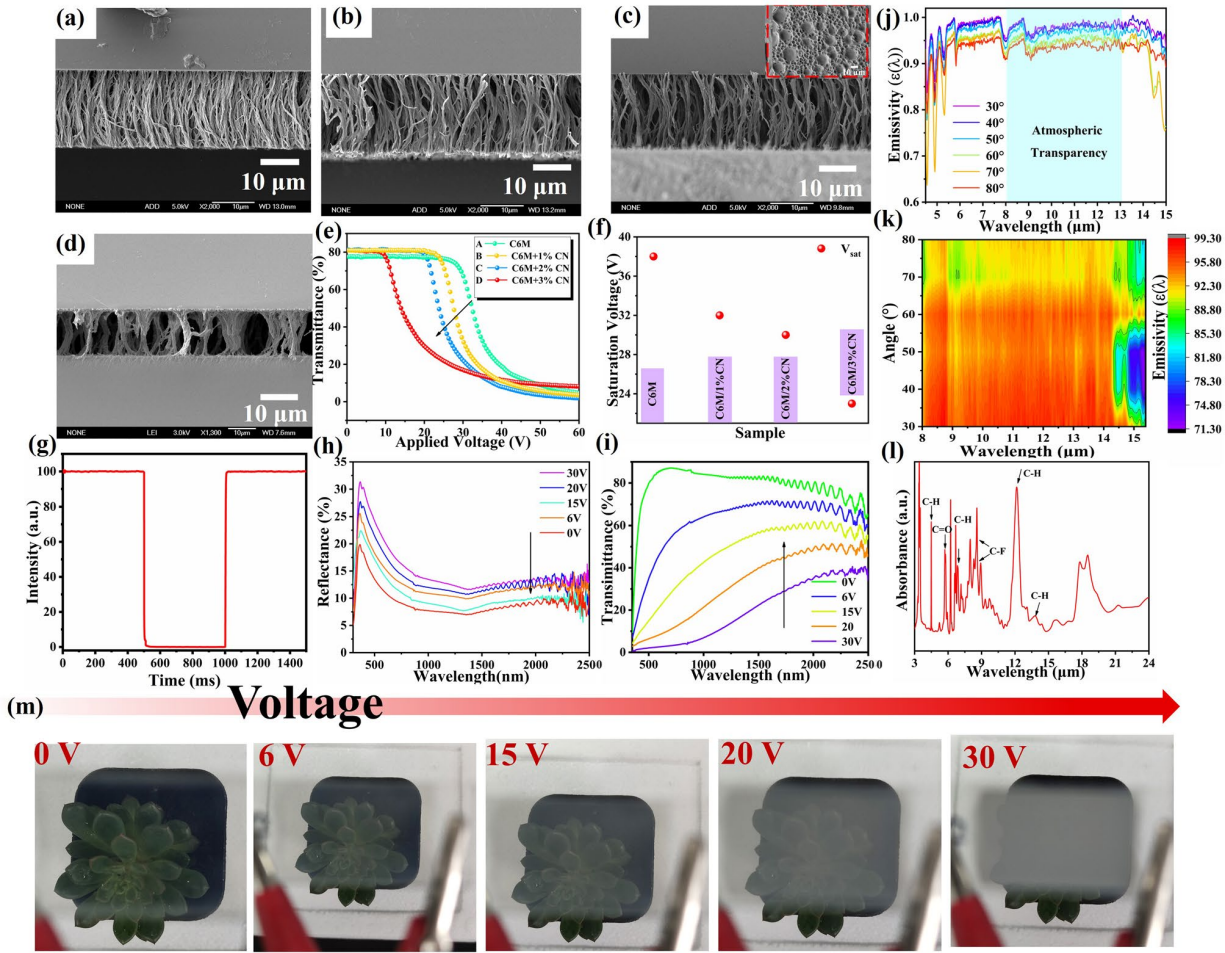
**a**–**d** SEM images of the polymer phase of PSLC films (inset shows the plane view); **e** E-O curve of the PSLC films; **f** saturation voltage variation trend; **g** transmittance-time curve of sample C; **h** reflectance spectra; **i** UV–Vis-Nir spectrum with voltage of sample C; **j** infrared emissivity spectra of a PSLC/PNIPAM film at different polarization angles (*θ*) from 30° to 80°; **k** measured polarization-dependent infrared emissivity spectra of the PSLC/PNIPAM film. **l** Absorbance spectrum of PSLC film with ATR-FTIR spectroscopy; **m** photographs of PSLC film transmittance at varying voltage levels

In the experiment, PSLCs are made from polymer and fluorine-containing negative LCs, which have high *ε*_LWIR_ and good radiative cooling efficiency because of their rich chemical bonds. To obtain a PSLC film with low switching voltage, monoacrylic monomer CN with cyano group was incorporated to modify the polymer network morphology. It has both mesogenic unit and flexible alkyl chains and exhibits good compatibility with LCs. The polymer morphology of the PSLC films (A–D samples) was studied using a scanning electron microscope, as illustrated in Fig. 4a–d. Sample information is provided in Table S1. It can be seen that in samples A–D, the upper and lower substrates are linked by vertical polymer dendrites. The increase of CN content results in both decreased polymer network density and increased pore size. When the concentration of CN monomer is maintained at 2.0 wt%, a uniform and stable membrane microstructure can be obtained. The inset image in Fig. 4c shows a plane view of the polymer. The electro-optical (E-O) curve of the PSLC film is shown in Fig. 4e. It can be observed that the E-O curve shifts significantly to the left with an increase in the CN content, and the saturation voltage decreases from 36 to 28 V, as shown in Fig. 4f. Figure 4g shows the transmittance-time curve of sample C, and a fast response in the millisecond range is presented. The CR of sample C was approximately 70, as shown in Fig. 4h. The reflectance and transmittance spectra of the films are shown in Fig. 4h, i. The reflectance increased with increasing voltage because the mismatch in the refractive indices between the LC and the polymer led to an increase in the scattering degree of the film. The transmittance curve decreased with increasing voltage, demonstrating dynamic control of light. Following this, the films were evaluated for their ability to regulate sunlight at different voltage levels, and the results are presented in Table 1. As depicted in Fig. 4i, it is possible to introduce multi-tiered adjustments to sunlight irradiation over the course of a day. The relevant parameters have been computed and are presented in Table 1. It has a very high transmittance at 0 V and the *T*_lum_ and *T*_sol_ values reached 79.7% and 76.2%, respectively. When the applied voltage reaches 30 V, the film exhibits low *T*_lum_ of 6.1% and *T*_sol_ of 50.3%. Images displaying various levels of light transmission are presented in Fig. 4m. It is evident that the film transmittance increases as the voltage decreases. Figure 4j–k shows the emissivity spectra of the PSLC/PNIPAM film in the infrared range of 4.0–15.0 µm at different polarization angles. Excitingly, the average emissivity across the atmospheric window exceeds 0.92 over a wide polarization angle range (30°–80°), indicating a stable heat flux through the atmospheric transparency window to the cold sink of outer space. The attenuated total reflection-Fourier transform infrared spectroscopy (ATR-FTIR) was used to observe the infrared spectrum of the thin film at 4–16 μm (as shown in Fig. 4l). The peak at 5.2–6.3 μm is attributed to the stretching motion of the C = O bond (*λ* = 5.2–6.3 μm, *v* = 1100–1380 cm^−1^), while the peak at 7.6–9.3 μm may be due to the bending vibration of the C–F bond (7.6–9.3 μm, 978–1280 cm^−1^). The peak at 11.7–14.5 μm is assigned to the bending vibration of the C-H bond (11.7–14.5 μm, 978–1037 cm^−1^). This provides a good explanation for the high emissivity of the film at 8–13 μm.

**Table 1:** Optical properties of PSLC film (20 μm) under different voltages

Transmittance	0 V	6 V	15 V	20 V	30 V
*T*_lum_ (%)	79.7	72.3	49.6	25.6	6.1
*T*_sol_ (%)	76.2	70.5	65.4	57.6	50.3
Δ*T*_lum_ (%)	–	7.4	30.1	54.1	73.6
Δ*T*_sol_ (%)	–	5.7	10.8	18.6	25.9

### Optical and Energy-Saving Demonstration of KPP Smart Window

**Fig. 5:**
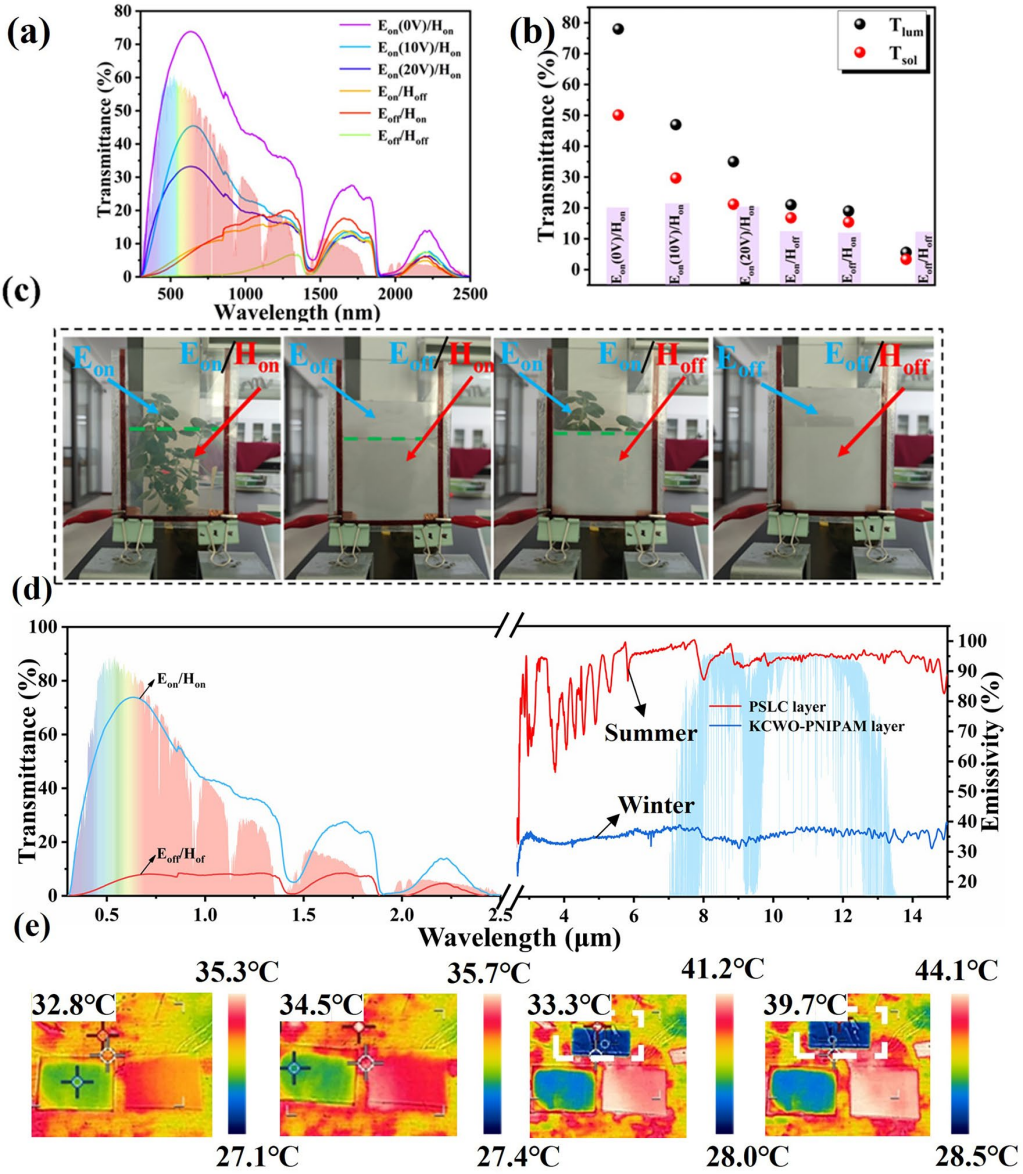
**a** Transmittance curves of KPP smart windows in different states; **b** *T*_sol_ and *T*_lum_ of composite windows in different states; **c** photographs of composite windows in different states; **d** solar transmittance in 0.3–2.5 μm and infrared emissivity curves in LWIR; **e** IR images for the low-E side (left) and high-E side (right) of KPP smart window at 34, 35, 41, and 44 °C, respectively (the IR image above is aluminum foil)

Two mechanisms: active control and passive control, are described in the preceding sections. However, neither of them alone can adequately address the requirements of smart windows in various environments. To enhance adaptability, a KPP smart window that features active–passive synergistic control was successfully constructed by integrating an active control PSLC layer with a passive control KCWO-PNIPAM layer. The PSLC layer was utilized as a foundation, and the surface of PET substrate was modified with polyvinylpyrrolidone. Subsequently, a polymerization reaction was employed to complete the integration. In comparison with single electrochromic and thermochromic smart windows, this design allows the adjustment of the window’s transmittance by applying voltage according to specific needs. When the temperature exceeds a certain threshold, the KCWO-PNIPAM layer undergoes a phase change, causing the window to become opaque. In addition, the adjustable εFront on both sides of the window can provide all-weather radiation cooling and heating effects tailored to weather conditions and individual preferences. Figure 5a displays the UV–VIS-NIR spectrum. It can be observed that the composite window in the on state has a maximum transmittance value of 78% in the visible light range, which meets the lighting requirements for daily use. In off state, the transmittance is only 4.2%, thereby providing excellent privacy. Simultaneously, it exhibits high absorption in the NIR range. Moreover, dynamic adjustment of the visible light is enabled by regulating voltage. The KPP smart window was evaluated for their ability to regulate sunlight at different voltage levels, and the results are presented in Table 2. Compared to a single PSLC layer, the *T*_lum_ value changes slightly, while the transmittance of NIR light experiences a significant reduction, thereby greatly enhancing the modulation capability in the presence of sunlight. Figure 5c shows the composite window’s modulation capability for sunlight in various states, with *T*_sol_ and *T*_lum_ of 52.6% and 78.4% in the on state, and 5.8% and 19.8% in the off state, respectively. As shown in Fig. 5c, the smart window allows for four distinct modes: a highly transparent state (*E*_on_, *H*_on_), an electrochromic state (*E*_off_, *H*_on_), a thermochromic state (*E*_on_, *H*_off_), and a highly opaque state (*E*_off_, *H*_off_). These functionalities are achieved through the collaborative interaction between the electrochromic properties of the PSLC layer and the thermochromic characteristics of the hydrogel layer. It demonstrates good privacy protection capabilities and the ability to provide multiple choices for distinct individual needs.

**Table 2:** Optical properties of KPP smart window under different voltages

Transmittance	0 V	6 V	15 V	20 V	30 V
*T*_lum_ (%)	78.4	68.3	47.6	23.6	5.8
*T*_sol_ (%)	52.6	34.5	27.4	23.6	19.8
Δ*T*_lum_ (%)	–	9.3	30	54	71.6
Δ*T*_sol_ (%)	–	14.7	21.8	25.6	29.4

On the other hand, as mentioned previously, *ε*_LWIR_ plays a vital role in energy saving. PSLC shows a very high emissivity (0.95) at the atmospheric window (8–13 μm) both in the on and off states because of its network structure and rich chemical bonds, indicating that it has a high radiative cooling efficiency. In contrast, the KCWO-PNIPAM has a low *ε*_LWIR_ of 0.35 (20 °C)–0.47 (40 °C), which is significantly lower than the normal glass (0.89). To further demonstrate the *ε*_LWIR_ difference of two sides of the KPP smart window, we compared its IR images with that of Aluminum foil at different temperatures (Fig. 5e). The bright white region is the high *ε*_LWIR_ side of the KPP smart window panel, because objects with high emissivity possess more intense thermal radiation. In contrast, the low-e surface appears blue (KCWO-PNIPAM and Al foil) in infrared images.

**Fig. 6:**
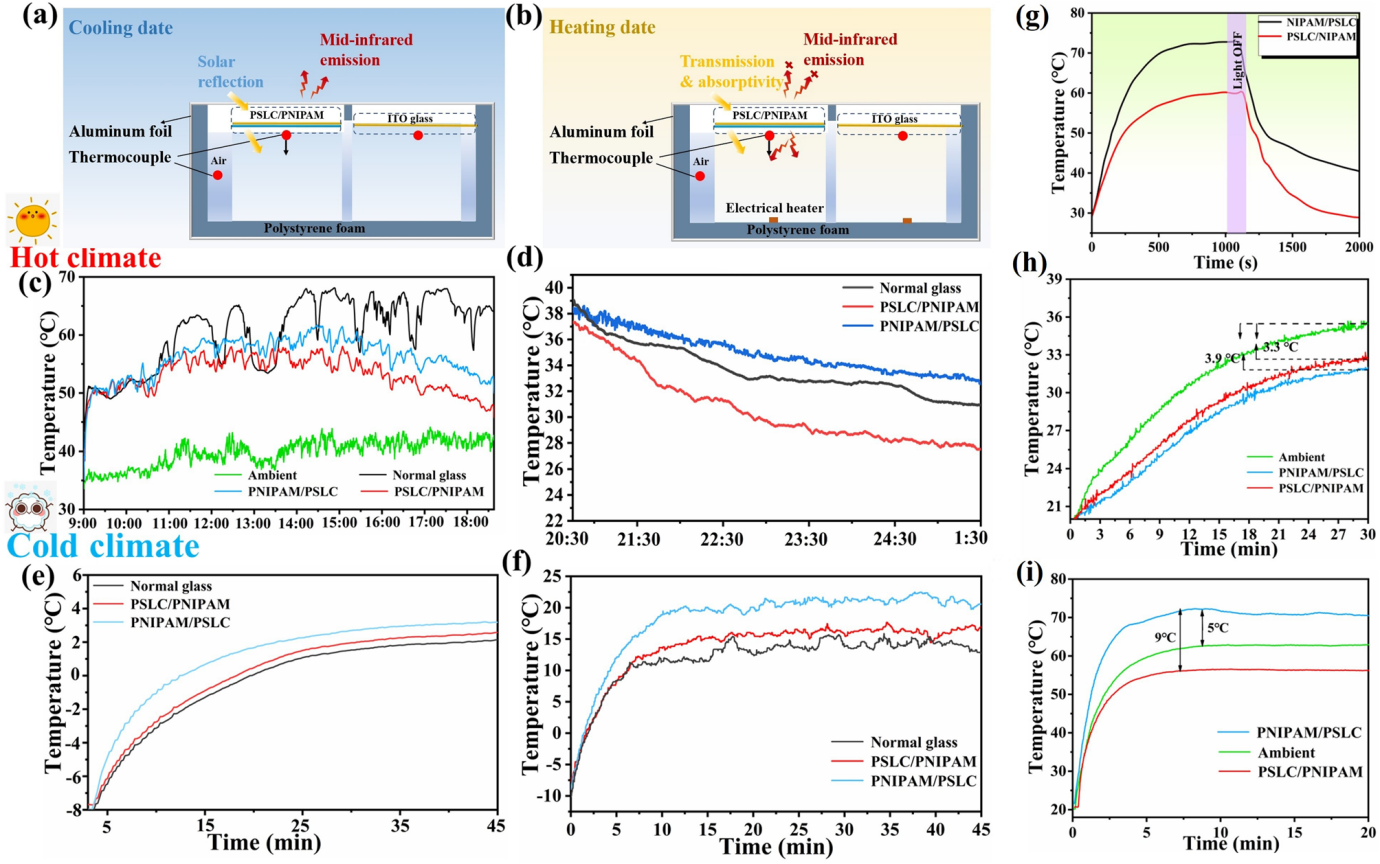
**a** Schematic representation of the apparatus utilized for evaluating cooling performance; **b** schematic representation of the apparatus incorporating a temperature control system for the evaluation of heating performance; **c**, **d** warming curves during the day and night; **e** air temperature for indoor cold environment comparative study for KPP window (high *ε*_Front_: PSLC/PNIPAM, low *ε*_Front_: PNIPAM/PSLC and normal glass, respectively. **f** Window inner surface temperature for the cold environment comparative study; **g** temperature curve with time at the simulated sunlight irradiation; **h** indoor air temperature; **i** outside surface temperature rising curves of the smart window

From the above discussion, it can be seen that the KPP smart window can spontaneously adjust the solar transmittance and switch the *ε*_Front_ through flipping the window to meet the energy-saving requirements in different seasons. In order to further verify its energy-saving performance, the temperature regulation performance of the KPP smart window was evaluated through outdoor hot and cold tests. The experimental device is illustrated in Fig. 6a, b. The sample container was a transparent acrylic chamber sealed with PE film. Three insulated polystyrene foam boxes wrapped with aluminum foil were placed in the acrylic chamber and covered with normal glass, KCWO-PNIPAM/PSLC (low *ε*_Front_) and PSLC/KCWO-PNIPAM (high *ε*_Front_), respectively. A K-type thermocouple was used to continuously record the real-time temperature of the sample and the ambient temperature inside the chamber. The ambient temperature inside the chamber was considered as the standard for evaluating the cooling performance of the KPP smart window. The continuous outdoor energy-saving performance evaluation was conducted in Beijing, China (39.87° N, 116.28° E) on July 7, 2024, as shown in Fig. 6a. PSLC/KCWO-PNIPAM, KCWO-PNIPAM/PSLC, and normal glass were installed on the box. Figure 6c shows the variation curve of the air temperature in the box. In this test, normal glass exhibited a maximum temperature of 48.2 °C. The maximum temperature of the PSLC/PNIPAM window was lowest among the three samples (43.5 °C). During the day, the temperature difference between the PSLC/PNIPAM window and normal glass was 4.7 °C (the corresponding solar irradiance and relative humidity are provided, as shown in Fig. S10). Figure 6d illustrates the cooling curve of the window during the night. It is evident that the PNIPAM/PSLC window exhibits the slowest cooling rate, maintaining a temperature that is 5 °C higher than that of a conventional window cavity. This observation highlights its excellent insulation properties. Figure S11 presents the results of the second outdoor performance test of the KPP smart window. Compared to the previous test, there were no significant changes in the results, indicating both good stability and the effectiveness of the experiment. Figure 6d shows the results of a simulated sunlight irradiation experiment. Under the same irradiation time, PNIPAM/PSCL exhibited a faster heating speed and a slower cooling rate, indicating better thermal insulation performance.

On the contrary, when the temperature was too low, the KPP smart window could be switched to the heating mode by flipping. The comparative experiment was conducted in a cold environment at 7 °C. The device used was the same as the above experimental device. A light bulb simulating sunlight was installed above the light bulb, and an electric heater was installed in the room to heat the indoor temperature. Figure 6e, f shows the changes in the indoor air temperature and the temperature of the inner surface of the window, respectively. PNIPAM/PSLC (low *ε*_Front_) has an air temperature of 2.5 °C. Meanwhile, PSLC/PNIPAM (high *ε*_Front_) has the air temperature of 2.1 °C. Besides, the temperature of the inner surface of the window in the cold environment experiment was also recorded. Compared with the air temperature, the difference in window surface temperature is more significant. PNIPAM/PSLC window (low *ε*_Front_) has the highest inner surface of 22.5 °C. Figure 6g shows the results of a simulated sunlight irradiation experiment. Under the same irradiation time, PNIPAM/PSCL exhibited a faster heating speed and a slower cooling rate, indicating better thermal insulation performance.

In order to study the thermal insulation performance test of KPP smart windows, a thermal insulation test simulation device was used, as shown in Fig. S9a. Figure 6h, i illustrates the curves of the KPP Smart Window surface temperature and indoor air temperature. Figure 6h shows the indoor air temperature rising curves of the window. It can be observed that, compared to ordinary windows, the indoor temperature of KPP smart windows increases at a slower rate, which indicates that the KPP smart composite hydrogel has excellent thermal insulation performance. Under the PNIPAM/PSLC state, the indoor temperature decreases by approximately 3.3 °C, while in the PSLC/PNIPAM state, it drops by about 33.9 °C. Figure 6i shows the outside surface temperature rising curves of the window. It has been observed that, under identical irradiation conditions, the surface temperature of PNIPAM/PSLC is 5 °C higher than that of ordinary glass. This increase is attributed to the excellent photothermal response of KCWO. In the PSLC/PNIPAM state, the temperature at the outer surface is approximately 4 °C lower than that of ordinary glass. The experimental findings mentioned above demonstrate that the smart window not only enables dual control by electricity and heat, but also exhibits outstanding thermal storage capabilities. It has good prospects for future applications. Moreover, this study assessed the electromagnetic shielding effectiveness of the KPP smart window (for details, see Section S7).

### Energy-Saving Potential Simulation

**Fig. 7:**
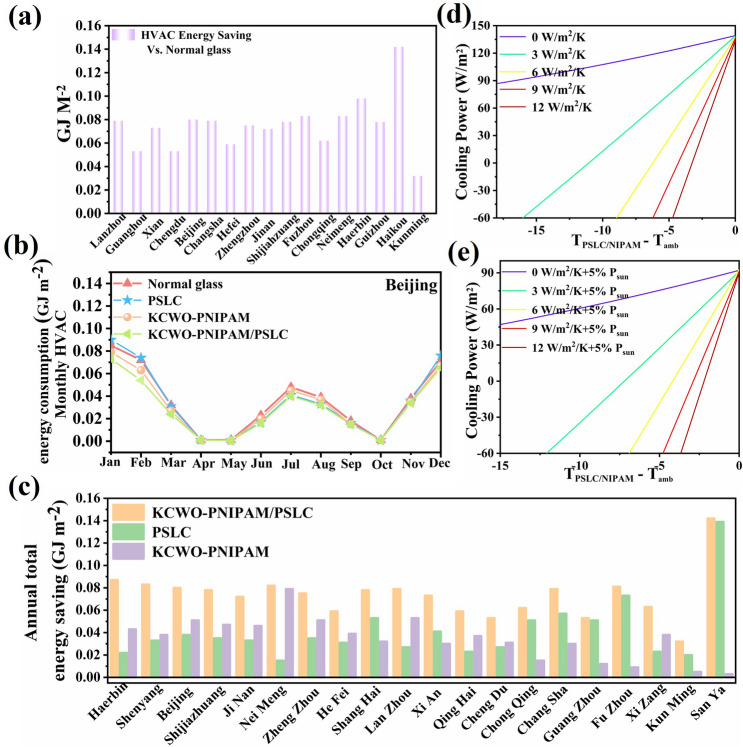
**a** Annual heating, ventilation, and air-conditioning (HVAC) energy saving for the KPP window against a normal glass as the baseline in the climate condition of 20 cities in China. **b** Monthly heating, ventilation, and HVAC energy consumption of normal glass, KCWO-PNIPAM, PSLC, and the KCWO-PNIPAM/PSLC window in the climate conditions of Beijing, **c** annual energy saving for the KCWO-PNIPAM window, PSLC, and KCWO-PNIPAM/PSLC window regarding the normal glass window in the climate conditions of 20 cities in China. **d** Calculated net cooling power of KCWO-PNIPAM/PSLC window during the nighttime; **e** calculated net cooling power of KPP window during the daytime

Energy PLUS software was used to simulate the energy-saving potential of the KPP smart window at cooling and heating modulation function (for details, see Section S8). The simulation was divided into heating season (November, December, January, and February), cooling season (June, July, and August), and transition seasons (the other months). The monthly HVAC energy consumption of the normal glass, KCWO-PNIPAM, PSLC and KPP smart window in cities with different climate conditions in China is shown in Figs. S13–S15 (Sect. S7). It can be found that in areas with strong solar radiation, PSLC and KPP smart windows (high *ε*_Front_) with higher emissivity show higher energy-saving efficiency. In contrast, in areas with weak solar radiation, the KCWO-PNIPAM and KPP smart windows (low *ε*_Front_) with lower emissivity have higher energy-saving efficiency. Figure 7b shows the HVAC energy consumption in Beijing for different months. In the cooling season, the KPP window and PSLC had lower energy consumption, whereas in the heating season, the KPP smart window and PNIPAM had lower energy consumption. Further simulation was conducted to estimate the annual HVAC energy-saving performance of the KPP smart window. With the normal glass as the baseline, the corresponding energy-saving map is shown in Fig. 7a, c. The theoretical analysis of the cooling power of the film was conducted using MATLAB (the relevant parameters were computed using Eqs. (S5–S9)). The ambient temperature was set to 303.15 K. The maximum cooling power of the PSLC/PNIPAM film during the night and day was 135.36 and 92.26 W m^−2^ K^−1^, respectively, as shown in Fig. 7d, e. In short, KPP windows can not only be used in different regions but also have very high energy-saving potential according to weather conditions in different seasons.

Finally, to illustrate the advantages of this study, a comprehensive comparison of electrochromic, thermochromic, and liquid crystal-based smart windows is presented from six perspectives: number of optical modes (NOM), adjustable solar spectral range (ASSR), adjustable emissivity (AE), the visible light transmittance variation (Δ*T*_lum_), switching speed (SR), and electromagnetic shielding effectiveness (ES). Compared to existing smart window technologies, this study demonstrates superior performance across multiple aspects, as shown in Fig. S16, with specific parameters detailed in Table S3. In summary, this research highlights the integration of optical performance and electromagnetic shielding functionality, which is of significant importance for the comprehensive application of smart windows in multifunctional fields (for details, see Section S8).

## Conclusion

In summary, we propose a smart window with actively adjustable radiative cooling and multi-level regulation abilities of sunlight. The smart window possesses different *ε*_LWIR_ at two sides (high *ε*_Front_ side: 0.95, low *ε*_Front_ side: 0.35), and is rationally designed to generate maximized radiative cooling in summer and good warm-keeping in winter, by combining the high *ε*_LWIR_ PSLC layer as the cooling side and the KCWO/PNIPAM layer as the heating side. PSLC not only has a fast response time of milliseconds but can also achieve multi-level regulation of solar transmittance by adjusting the applied voltage, ensuring high radiative cooling efficiency to meet the needs of use in hot seasons. Additionally, it can easily switch from cooling to heating during cold weather while maintaining excellent heat retention. As a photothermal nanoconverter, KCWO efficiently and stably absorbs sunlight to provide continuous heat for smart windows. Meanwhile, the lower *ε*_LWIR_ and higher specific heat capacity of KCWO/PNIPAM provide good thermal insulation properties. Outdoor experimental tests showed that compared with ordinary glass, PSLC/PNIPAM exhibits a maximum temperature drop of 12.3 °C. In addition, when tested in a cold environment, the indoor temperature of PNIPAM/PSLC is significantly higher than that of ordinary glass. Meanwhile, it has also shown good efficiency in the field of electromagnetic shielding. The proposed composite system is a promising candidate for developing smart energy management devices.

## Supplementary Information

Below is the link to the electronic supplementary material.

Supplementary file1 (DOCX 6343 KB)
