# Supplementary material for: A Janus Smart Window for Temperature-Adaptive Radiative Cooling and Adjustable Solar Transmittance
Source: Nanomicro Lett. 2025 Apr 27;17:233. doi: 10.1007/s40820-025-01740-1 (PMC12033136; doi:10.1007/s40820-025-01740-1)
Supplement: Supplementary file 1 — Supplementary file1 (DOCX 6343 KB) [file 40820_2025_1740_MOESM1_ESM.docx]

Supporting Information for

**A Janus Smart Window for Temperature-Adaptive Radiative Cooling and Adjustable Solar Transmittance**

Zuowei Zhang^1^, Meina Yu^1^*, Cong Ma^3^, Longxiang He^1^, Xian He^1^, Baohua Yuan^1^ Luoning Zhang^1^, Cheng Zou^1,2^*, Yanzi Gao^1^, Huai Yang^1,2,3^*

^1^Institute for Advanced Materials and Technology, University of Science and Technology Beijing, Beijing 100083, P. R. China

^2^Beijing Advanced Innovation Center for Materials Genome Engineering, University of Science and Technology Beijing, Beijing 100083, P. R. China

^3^School of Materials Science and Engineering, Peking University, Beijing 100871, P. R. China

*Corresponding authors. E-mail: [yumeina@ustb.edu.cn](mailto:yumeina@ustb.edu.cn) (Meina Yu); [zoucheng@ustb.edu.cn](mailto:zoucheng@ustb.edu.cn) (Cheng Zou); yanghuai@pku.edu.cn (Huai Yang)

**S1 Schematic Concept of the Designed Smart Window**


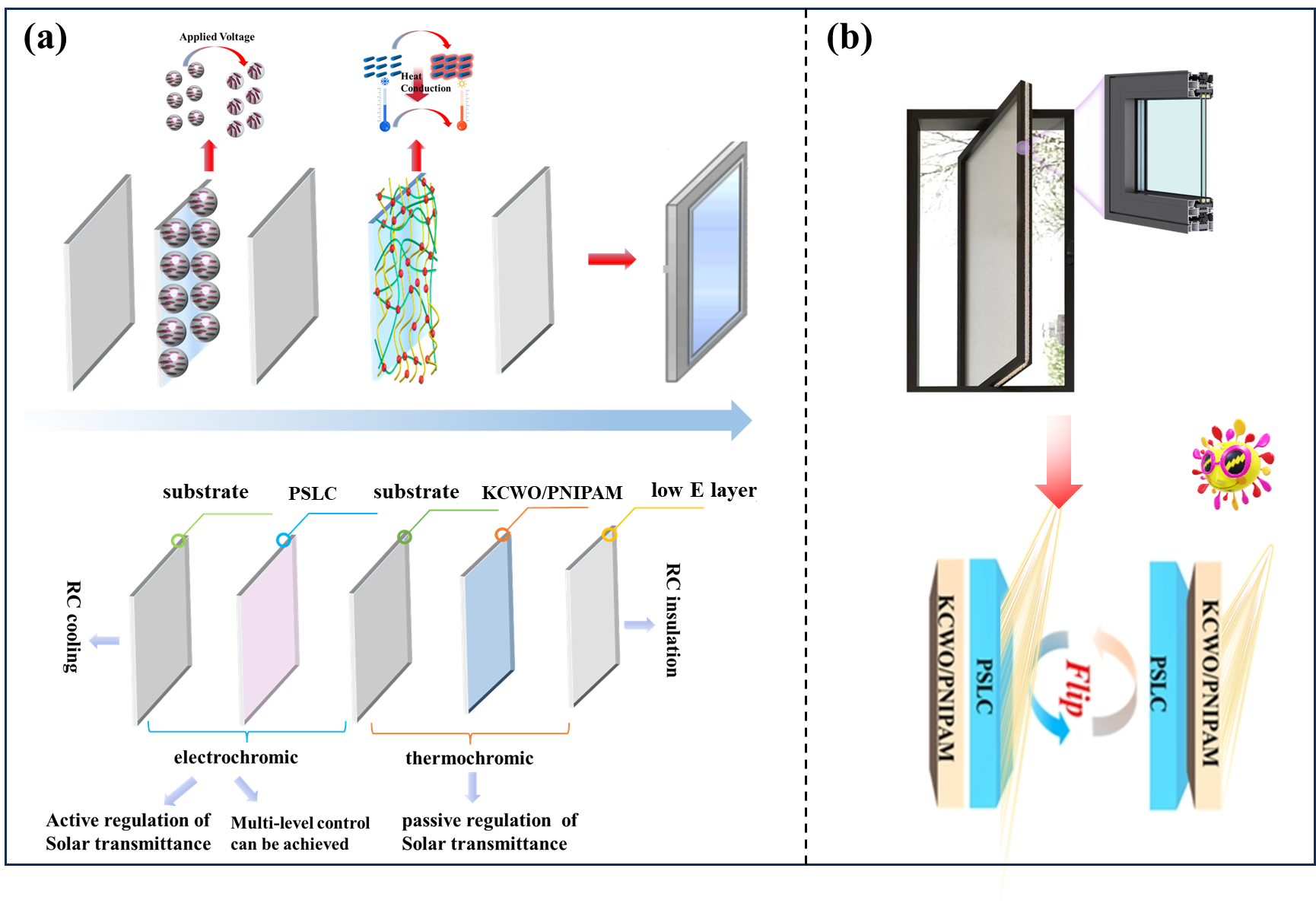


**Fig. S1** (**a**) Scheme of the structure for Janus smart window panel; (**b**) The schematic illustration for the fabrication and modulation mechanism of smart windows

**S2 Experimental**

**S2.1 Materials**

The negative nematic liquid crystal GXV-7822-180 (TNI = 380.15 K, Δε = −4.4) was purchased from Jiangsu Hecheng Display Technology Co., Ltd. The liquid crystal monomers 2-methyl-1,4-phenylene bis(4-((6-(acryloyloxy)- hexyl) oxy) benzoate) (C6M) and Cyano mono-acrylate monomer (CN) were purchased from shanghai Meryer Chemical Technology Co., Ltd. IR 651 was purchased from Bide Pharmatech Ltd., Shanghai, China. Tungsten trioxide (WO_3,_ AR, 98.0%), Potassium nitrate (KNO_3_, AR, 99.5%) and cesium carbonate (Cs_2_CO_3_, AR, 99.9%) were bought from shanghai Macklin Biochemical Technology Co., Ltd. Trioxide (WO_3_, AR, 99.0%), lithium nitrate (LiNO_3_, AR, 99.5%), N, N-methyl-enebisacrylamide (C_7_H_10_N_2_O_2_, AR, 99.0%) and N-isopropylacrylamide (C_6_H_11_NO, AR, 98%) were bought from shanghai Meryer Chemical Technology Co., Ltd.


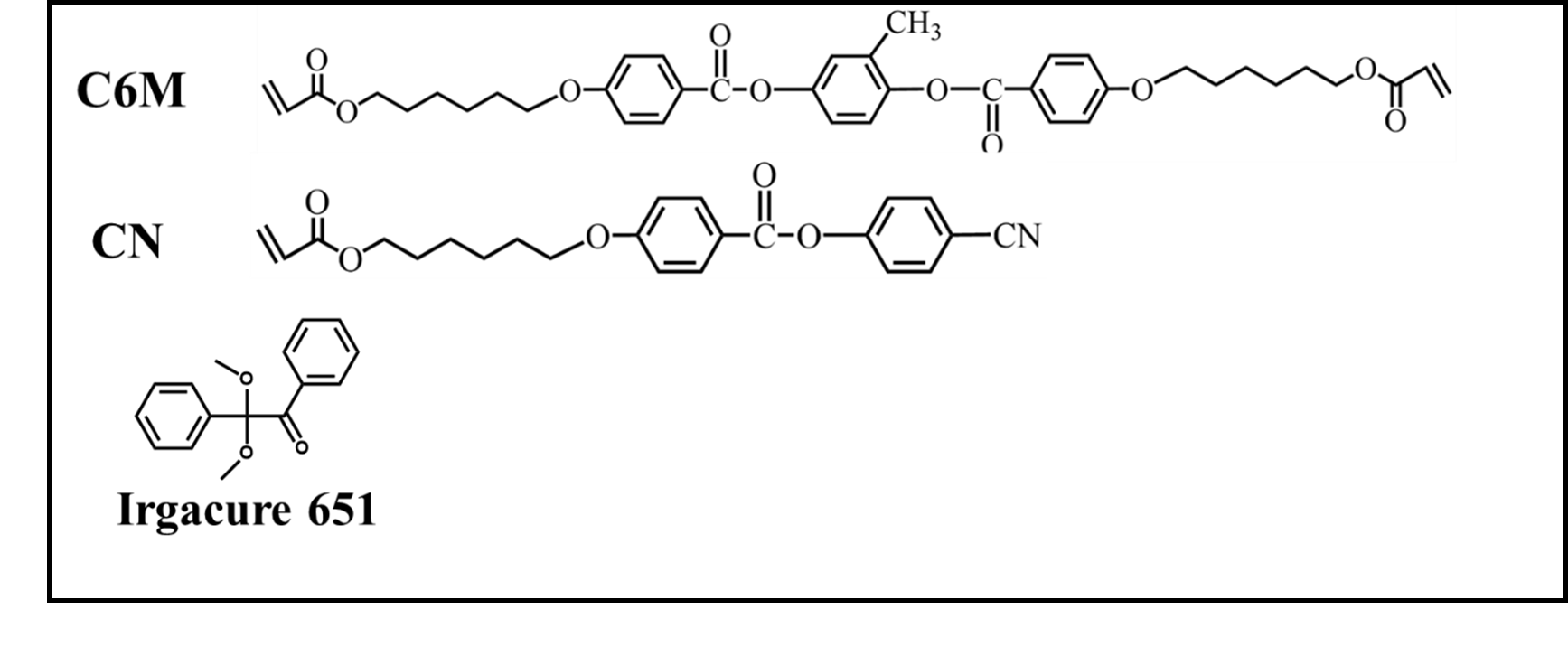


**Fig. S2** Molecular structures of monomers and photo-initiators

**Table S1** Compositions and reaction conditions of each sample used in this study

| Sample | Compositions | Weight Ratio (wt%) | GXV-7822-180 (wt%) |
| --- | --- | --- | --- |
|  | | | |
| A | C6M/CN/651 | 7.5/0/0.1 | 92.4 |
| B | C6M/CN/651 | 6.5/1/0.1 | 92.4 |
| C | C6M/CN/651 | 5.5/2/0.1 | 92.4 |
| D | C6M/CN/651 | 4.5/3/0.1 | 92.4 |

**S2.2 Synthesis of K_m_Cs_n_WO_3_ nanoparticles**

The specified quantities of tungsten trioxide, potassium nitrate, and cesium carbonate were measured and thoroughly blended in a molar ratio of n (K + Cs) / n (W) equal to 0.35. The resulting mixture was subjected to calcination at 700℃ in a hydrogen (H_2_) atmosphere for a duration of 40 minutes. Following this, the powders of K_m_Cs_n_WO_3_ (KCWO) were produced upon cooling naturally to ambient temperature.

**S2.3 Prepared of KPP smart window**

First, LCs, monomers, photo-initiator, and spacers are mixed and uniformly coated on top of the PET/ITO layer in a specific ratio (The monomer ratio and chemical structure are shown in Table S1 and **Fig.** S2.). Subsequently, they are polymerized into a film in situ through ultraviolet light irradiation. Hydrophilization of PET surface with polyvinyl pyrrolidone, Afterwards, the KCWO powder obtained was dispersed in a polyvinyl alcohol (PVA) solution at a weight concentration of 5% and stirred at 45℃ for 40 minutes. Subsequently, transfer it to the PET and PE sandwich, and the polymerization reaction proceeded at 66 ℃ for 6 hours with potassium persulfate (KPS) as the initiator. After the reaction finished, the PVA-KCWO/PNIPAM dual network inter-transfer hydrogel layer was successfully prepared. Ultimately, a smart window device with electro and thermal dual responsiveness was assembled in a sandwich structure, as depicted in **Fig. S1**. The samples contain different concentrations of KmCsnWO_3_ in the hydrogel, with (KCWO) = 0.004g/mL, 0.008g/mL, 0.012g/mL, 0.016g/mL and 0.02g/mL, respectively.

**S2.4 Characterization**

The phase compositions of the samples were determined by X-ray diffraction analysis (XRD, Shimadzu XRD-7000S). Surface morphology of the samples was observed using scanning electron microscopy (SEM, JSM-7800). The Fourier transform infrared spectrometer (PerkinElmer Lambda FTIR-7600) was used to analyze the functional groups present in the samples. High-resolution transmission electron microscopy (HRTEM, JEM-2100) was used to analyze the microscopic morphology and lattice structure of the samples. The chemical compositions were observed using energy dispersive spectroscopy (EDS). The chemical composition and chemical environment were analyzed using X-ray photoelectron spectroscopy (XPS, Perkin-Elmer PHI 5000C). The temperature changes of a smart window surface irradiated by infrared light were determined using infrared thermography (FLIR E4). Furthermore, to assess the dimming capability of the PDLC devices, the transmission spectra of the smart window were recorded using a UV-Vis-NIR spectrophotometer (PerkinElmer Lambda 950). The integral luminous transmittance (T_lum_, 380 nm-780 nm) and solar transmittance (T_sol_, 300 nm-2500 nm) were calculated from the transmittance spectrum of the film using the following equations:

$Tlum=\int\varphi lum\left( \lambda\right)T\left( \lambda\right)d\lambda/\int\varphi lum\left( \lambda\right)d(\lambda)$ (S1)

$Tsol=\int\varphi sol\left( \lambda\right)T\left( \lambda\right)d\lambda/\int\varphi sol\left( \lambda\right)d(\lambda)$ (S2)

Where *T*(λ) denotes the spectral transmittance, and $\varphi$lum(λ) is the standard luminous efficiency function of photopic vision in the wavelength range 380-780 nm. $\varphi$sol(λ) is the solar irradiance spectrum for air mass 1.5 corresponding to the sun standing 37 ℃ above the horizon [S1-S3]. The visible light transmittance variation (ΔT_lum_), and the solar light modulation ability (ΔT_sol_) are obtained by the following formula:

$\Delta Tlum=Tlum\left( on \right)-Tlum(off)$ (S3)

$\Delta Tsol=Tsol\left( on \right)-Tlsol(off)$ (S4)

The photothermal and thermal insulation performance of the PDLC composite window was evaluated using a custom thermal insulation testing apparatus.

The emissivity spectra within the wavelength range of 2.5 to 14 μm were examined through Fourier transform infrared spectroscopy (FTIR) utilizing the Nicolet iS50 instrument from the United States. As per Kirchhoff's law, it is anticipated that the spectral absorptivity (𝛼 (𝜆)) and emissivity (ε(𝜆)) should be equal for an object in thermal equilibrium. Consequently, the emissivity (*ε* (𝜆)) was calculated as *ε* (𝜆) = 1−(𝜆)−𝜏(𝜆), where 𝜌(𝜆) and 𝜏(𝜆) represent the reflectance and transmittance value, respectively. The values in the long-wave infrared (LWIR) region spanning from 8 to 14 μm were determined using a dual-band emissivity measurement apparatus (IR-2). For each specimen, the LWIR values were measured at six distinct locations, and the mean value was regarded as the ultimate LWIR value. The attenuated total reflection Fourier transform infrared (ATR FTIR) assessments were carried out employing a Nicolet 6700 spectrometer.

The cooling capacity calculations for PRC films are influenced by solar irradiance and atmospheric downward thermal radiation under clear sky conditions. Heat transfer takes place between the PRC films and the surrounding environment, in line with the principle of energy conservation. The net cooling power (P_net_) can be mathematically represented by the following equation [S2-S4].

$P_{net}\left( T \right)=P_{rad}\left( T \right)-P_{atm}\left( T \right)-P_{sun}-P_{cond+conv}$ (S5)

The variable P_rad_(T) represents the radiation power emitted from the surface of a radiative cooler in a specific hemisphere, and is determined through a defined mathematical formula:

$P_{rad}\left( T \right)=A\int d\Omega cos\theta\int_{0}^{\infty} d\lambda I_{BB}\left( T,\lambda\right)\varepsilon\left( \lambda,\theta\right)$ (S6)

Where $\int d\Omega=2\pi\int_{0}^{x} d\theta sin\theta$ represents the angular integral across a hemisphere; The spectral distribution of thermal energy emitted by a blackbody at temperature T is denoted by $I_{BB}(T,\lambda)=\frac{2hc^{2}}{\lambda^{5}}\frac{1}{e^{hc/(\lambda k_{B}T)}-1}$. In this context, *h* represents Planck's constant, *k_B_* signifies the Boltzmann constant, *c* denotes the speed of light, and 𝜆 stands for the wavelength. The term *ε* (𝜆, 𝜃) refers to the spectral and angular emissivity at temperature T. A represents the surface area of the radiative cooler.

$P_{atm}\left( T_{amb} \right)=A\int d\Omega cos\theta\int_{0}^{\infty} d\lambda I_{BB}\left( T_{amb,}\lambda\right)\varepsilon\left( \lambda,\theta\right)\varepsilon_{atm}\left( \lambda,\theta\right)$ (S7)

P_atm_ (T_amb_) represents absorbed power due to incident atmospheric thermal radiation.

$\varepsilon_{atm}(\lambda,\theta)=1-\tau(\lambda)^{1/cos\theta},$ where 𝜏(𝜆) is the atmospheric transmittance in the zenith direction.

$P_{solar}=A\int_{0}^{\infty} d\lambda\varepsilon\left( \lambda,\theta_{solar} \right)I_{AM1.5}\left( \lambda\right)$ (S8)

The P_solar_ value signifies the energy absorbed by the film from incoming solar radiation, where I_AM1.5_(𝜆) represents the standard solar irradiance.

$P_{cond+con\nu}\left( T,T_{amb} \right)=Ah_{c}\left( T_{amb}-T \right)$ (S9)

where h_c_ is combined conduction and convection heating, which is set as 0, 3, 6, 9 and 12 W m^−2^ K^−1^ in this study.

**S3 Characterization of KCWO/PNIPAM Hydrogel**

**
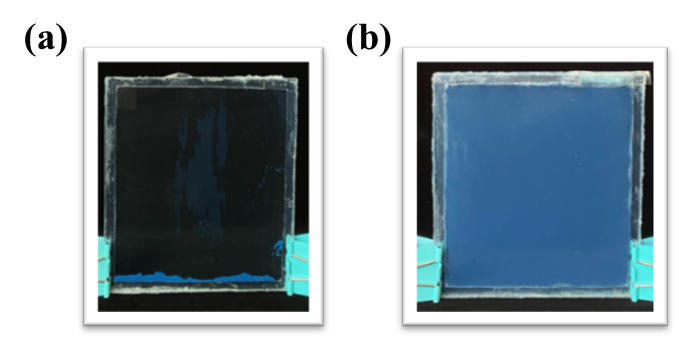
**

**Fig. S3** (**a**) KCWO/PNIPAM composite hydroge window, (**b**) PVA-PNIPAM/ KCWO composite hydrogel window prepared by modifying KCWO particles with PVA

**Figure S3** shows the actual photos of KCWO/PNIPAM and PVA- KCWO/PNIPAM composite hydrogel windows. It can be found that the KCWO particles in the KCWO/PNIPAM hydrogel window have precipitated after multiple phase transitions, while the PVA- KCWO/PNIPAM composite hydrogel exhibits improved stability after the KCWO particles being modified by PVA.

**
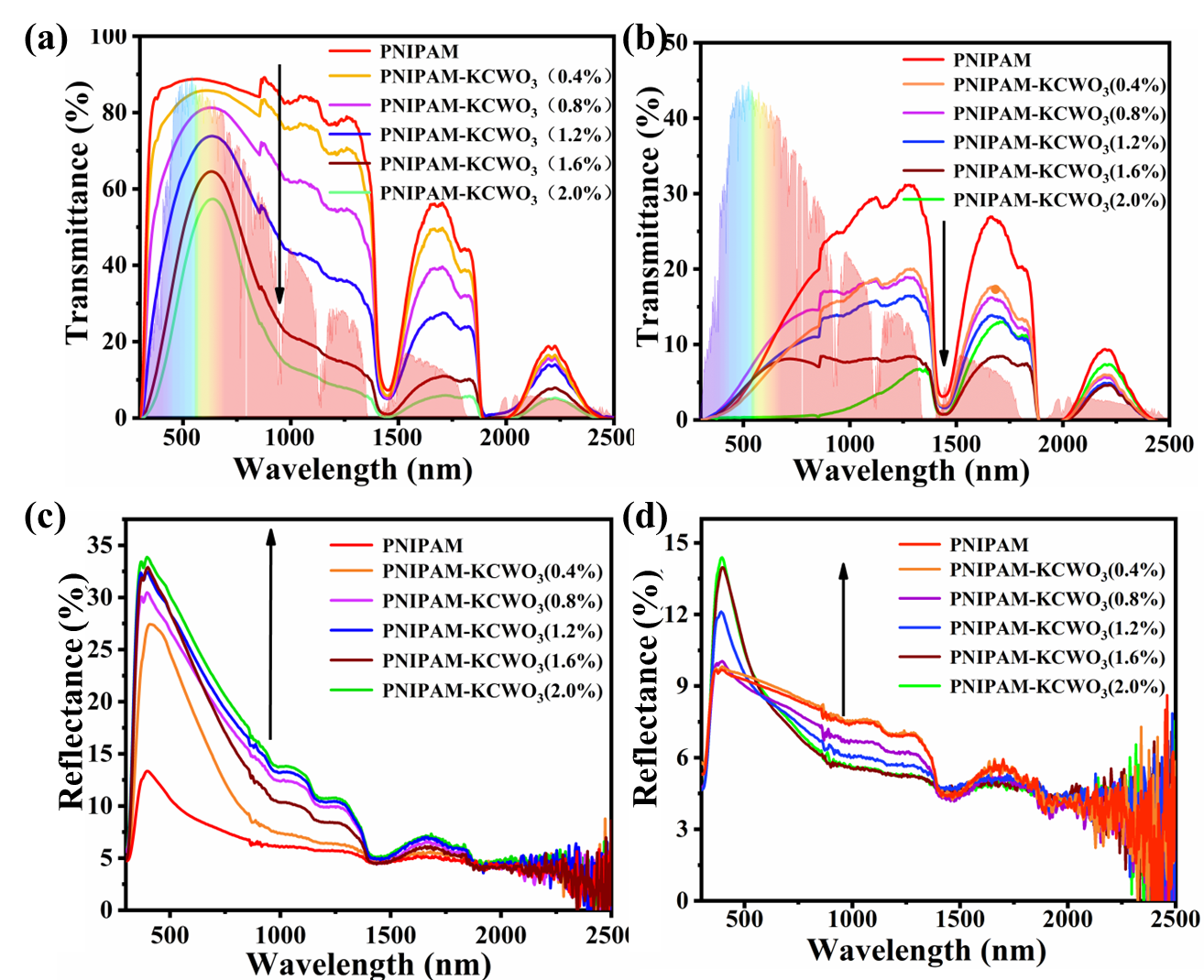
**

**Fig. S4** The transmittance and reflectance of PNIPAM at full solar spectrum with different mass fractions of KCWO at 25 ℃ and 40 ℃

**S4 Stability of the Composite Hydrogel Layer and PSLC Layer**


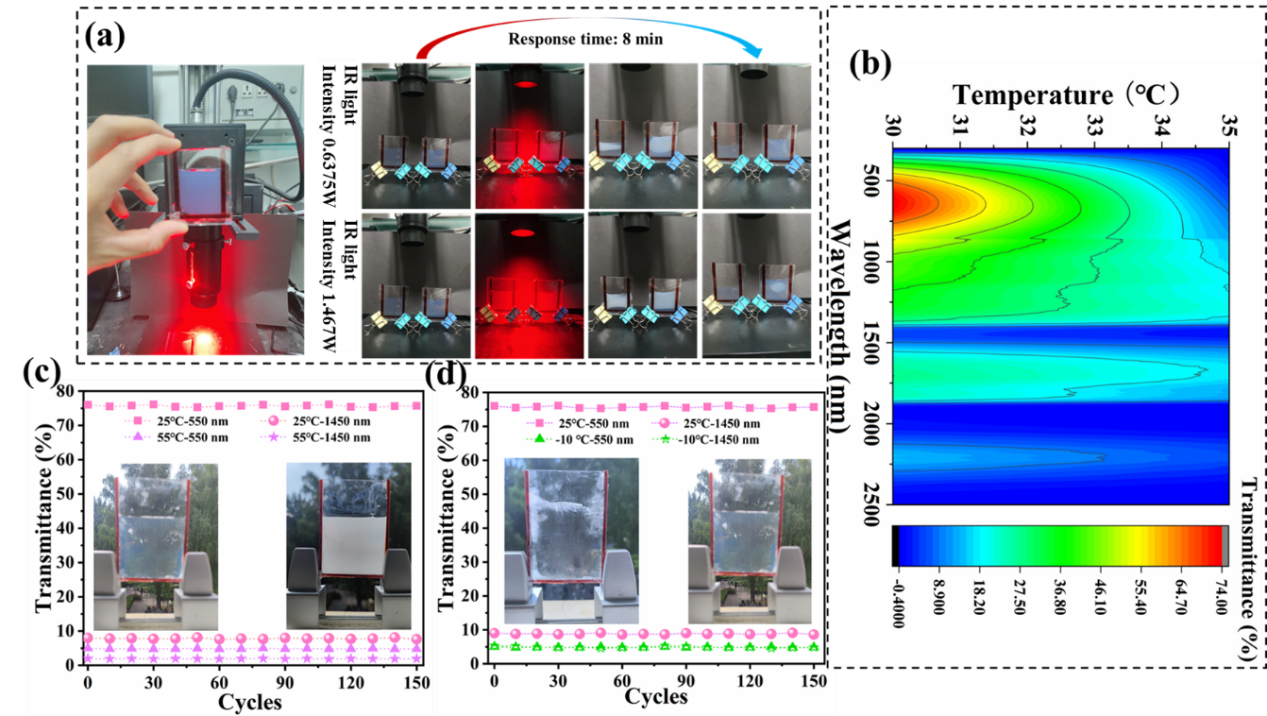


**Fig. S5** (**a**) Response time test of the KCWO/PNIPAM composite hydrogel layer under irradiation of infrared light with different intensity; (**b**) the UV-Vis-NIR spectra at different temperature; (**c**) Cycling stability performance test at high temperature and (**d**) cycling stability performance test at low temperature

The low-E glass utilized in this context is ITO glass, which exhibits lower resistance compared to FTO glass, thereby facilitating a reduction in driving voltage. More importantly, employing ITO as the conductive layer enhances transmittance. Given that the KPP smart window comprises multiple layers, ITO glass is selected to ensure optimal transmittance of the window.





**Fig. S6** Comparison of Transmittance between ITO and FTO

We investigated the impact of the thickness of PSLC layer films on sunlight transmittance and the variations in internal temperature of the films under simulated sunlight exposure, as illustrated in the **Fig. S7**. It is evident that as the film thickness increases, the transmittance at wavelengths ranging from 300 to 2500 nm decreases. However, the radiative cooling effect is enhanced. In comparison to a 20 μm thick film, the 50 μm thick film exhibited superior cooling performance, with the internal temperature of the film decreasing by approximately 4.8 ℃ after 800 seconds of exposure.


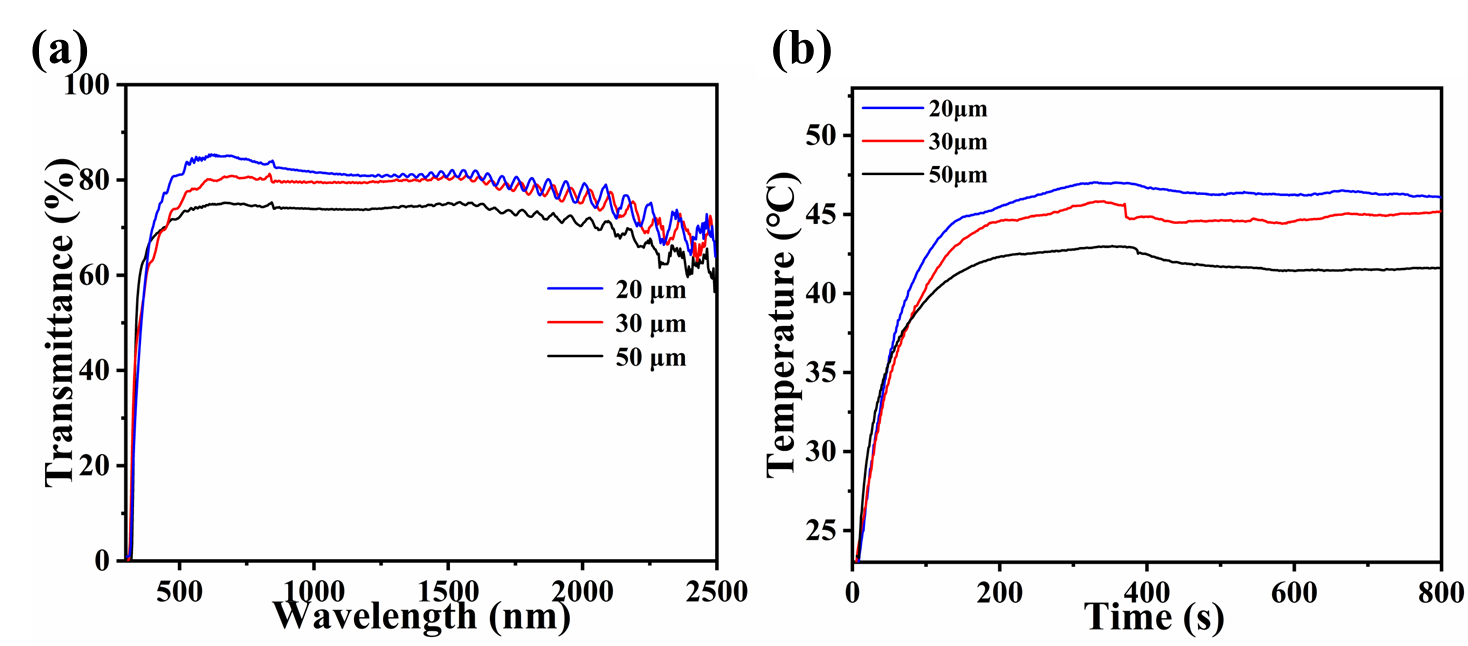


**Fig. S7** (**a**) UV-Vis-NIR transmission spectra of films in different thickness; (**b**) The variation in indoor temperature of films with different thicknesses

**Figure** S8a shows transmittance variation of PSLC film during cycling test and the transmittance in the transparent and opaque states is almost constant. Then, the transmittance spectra of the film before and after the cycles were compared as shown in Fig S8b, the transmittance spectra of film after 500 cycles were almost unchanged compared to the original film, indicating good durability and switchability of the film.

*
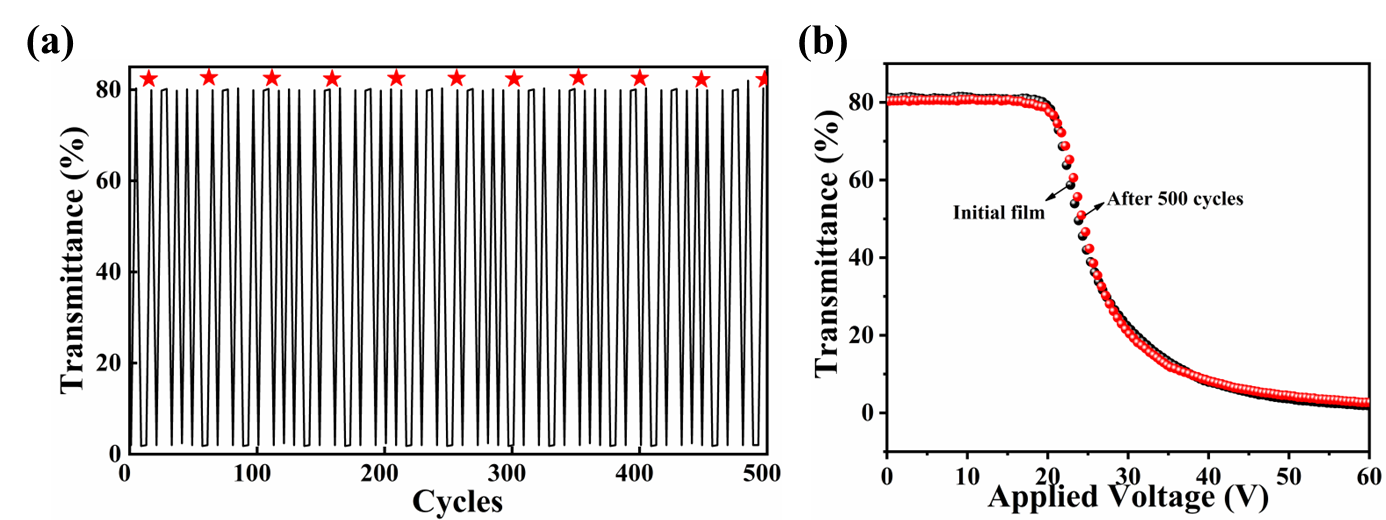
*

**Fig. S8** (**a**) transmittance variation of PSLC film during cycling test; (**b**) the transmittance of the spectra of the initial film and the film after 500 cycles

**S5 Thermal Insulation Performance Test**


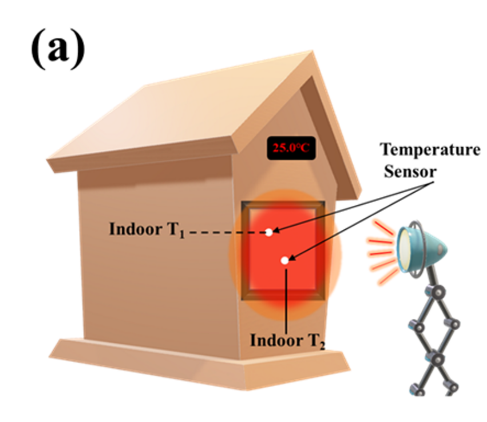


**Fig. S9** Window experiment simulation diagram





**Fig. S10** Solar irradiance, wind speed, and relative humidity for outdoor experiment

**

**

**Fig. S11** Warming curves during the day

**S6 Electromagnetic Shielding Properties**

In addition to regulating visible light, near-infrared light, and mid-to-far infrared wavelengths, the ability to control electromagnetic waves is crucial for the development of future smart windows. Therefore, the efficiency of electromagnetic shielding (Within the X-band frequency range (8.2–12.4) GHz) is investigated, and enhanced performance from 22 to 28 is achieved by modifying the transparent conductive electrode.

The scattering parameters (S_11_ and S_12_ or S_21_ and S_22_), the relative complex permittivity (εr = ε′ − jε′′) and the complex permeability (μr = μ′ − jμ′′) were measured by Agilent PNAN5244A vector network analyzer (Agilent Technologies, Inc., Palo Alto, CA, USA) within frequency range of X-band using waveguide method. All custom paper samples (22.86 mm × 10.16 mm) were fixed with copper sample clamp to ensure the connection between the waveguide flanges. The reflection coefficient (R) and transmission coefficient (T) were determined by the S parameters (S_11_, S_12_, S_21_, S_22_). There was an equilibrium between R, T, and the absorption coefficient (A), which could be calculated using the following formula [S9–S11]:

R=$\left| s_{11} \right|^{2}$= $\left| s_{12} \right|^{2}$ (S10)

T=$\left| s_{12} \right|^{2}$= $\left| s_{21} \right|^{2}$ (S11)

R+T+A=1 (S12)

Schelkunoff's theory posits that the total electromagnetic interference scattering efficiency (SE_T_) is the aggregate of surface reflection loss (SE_R_), multiple reflection loss (SE_M_), and absorption loss (SE_A_) occurring within the material. This relationship can be articulated through the following equation [S27, S32–S34]:

SE_T_ (dB)=-10$\log\left( \frac{PT}{p_{I}} \right)=\mathrm{SE}_{R}+\mathrm{SE}_{A}+\mathrm{SE}_{M}$ (S13)

SE_R_ (dB)=-10$\log\left( 1-s_{11}^{2} \right)= -10\log\left( 1-s_{12}^{2} \right)$ (S14)

$\mathrm{SE}_{A}\left( \mathrm{dB} \right)=-10log \left( \frac{s_{12}^{2}}{1-s_{11}^{2}} \right)=-10log \left( \frac{s_{12}^{2}}{1-s_{11}^{2}} \right)$ (S15)

There are studies that show that the SE_M_ could be neglected when the SE_T_ > 15 dB and it could be described as [S12–S14]:

$\mathrm{SE}_{T} \approx\mathrm{SE}_{R} +\mathrm{SE}_{A}$ (S16)

The electromagnetic characteristics of film within the X-band frequency range were evaluated using a waveguide cavity. The findings from these tests are presented in **Fig. S11**. Specifically, **Fig.** S12(a) illustrates the reflection coefficient (R) curve associated with the different transparent conductive electrode. The results of the absorption coefficient A curve of film are shown in **Fig.** S12(b). It can be observed that as the frequency of an electromagnetic wave increases, the absorption coefficient (A) is inversely related to the reflection coefficient. The shielding efficiency of window is calculated according to formulas (S13) - (S15). The results are shown in **Fig.** S12c (reflection loss SE_R_), **Fig.** S12d (absorption loss SE_A_), and **Fig.** S10e (the overall shielding performance SE_T_ of ITO and Ag NWS material).

**
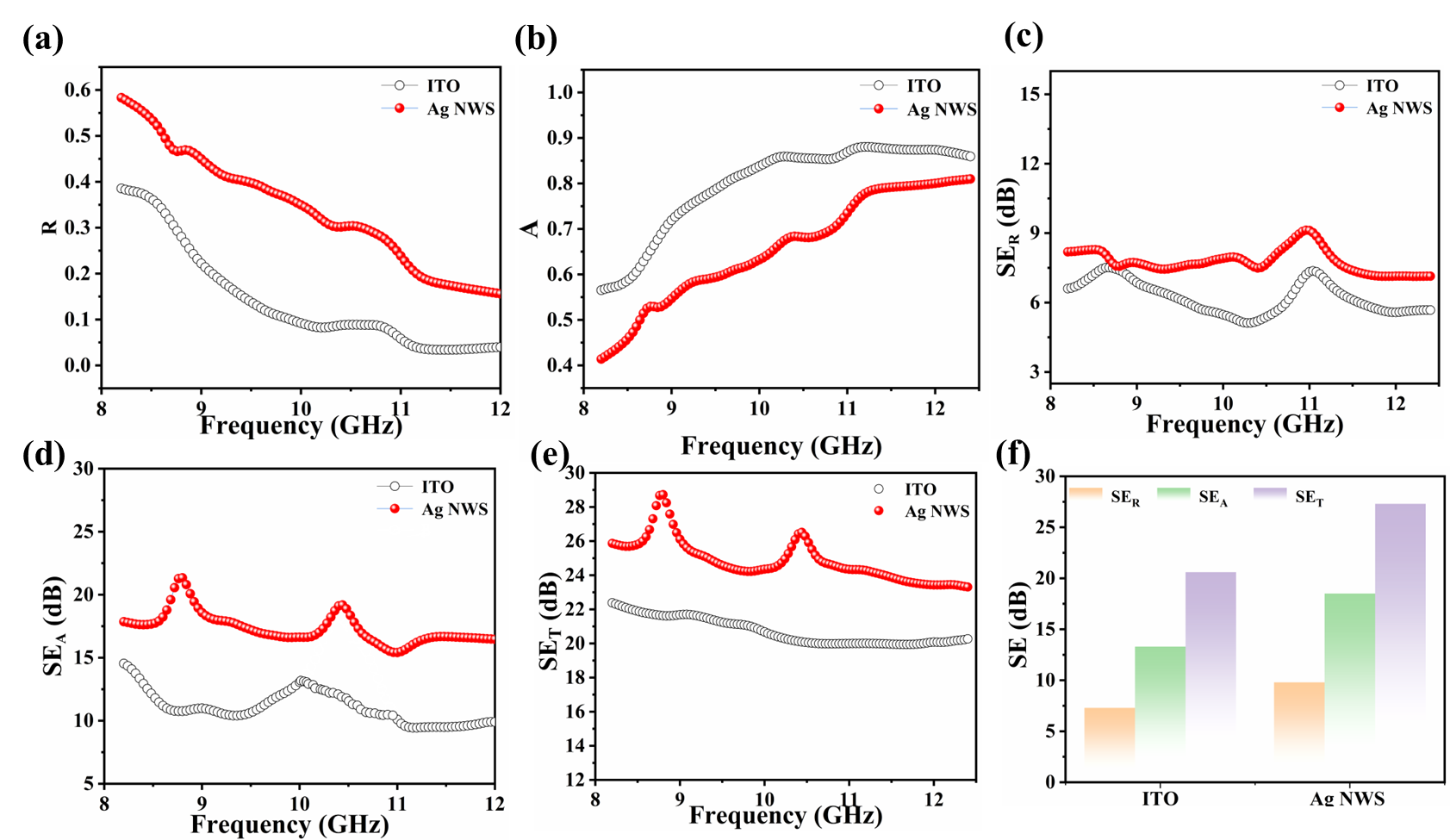
**

**Fig. S12** KPP window in the X-band (**a**) reflection coefficient R, (**b**) absorption coefficient A, (**c**) reflection loss SE_R_, (**d**) absorption loss SE_A_, (**e**) overall shielding performance SE_T_, and (**f**) average EMI SE of the transparent conductive electrode material (Arrows show an overall upward trend).

**S7 Energy-Saving Potential Simulation**

The energy consumption of the smart windows was evaluated using the Energy Plus software. A model of a 10-story office building was built for simulation testing with floor dimensions of 40 m (131 ft, L) × 40 m (131 ft, W) × 4 m (13 ft, H). The total window area of the glazing system in the building was 4,636 square meters, and the windows accounted for 40% of the total wall area [S5-S6]. The lighting control scheme in the Energy Plus model was created by a DOE built using a prototype building energy simulation model. Daylighting controls were incorporated into the lighting control model by implementing daylighting reference points in all perimeter thermal zones and core thermal zones. In addition, the average power density was set to 8.50 W m^− 2^ based on the zone floor area in accordance with the ASHRAE standards and guidelines [S7-S8]. The detailed optical properties of the samples used to simulate the cooling and heating energy consumption are summarized in Table S2. June, July, and August are the cooling seasons; November, December, January, and February are the heating seasons, and the other seasons are transition seasons, where 20 and 26 ℃ were adopted as the internal boundary conditions for heating and cooling, respectively. Hourly weather data for typical meteorological years (TMY) was used as the external boundary conditions. Energy efficiency is defined as the difference between various specimens and ordinary glass. This model adheres to energy efficiency codes and standards, utilizing a typical operation and occupancy schedule, standard lighting and miscellaneous loads, and HVAC systems. The electrochromic and thermal-to-chromic performances of the samples were controlled by the built-in EMS function of Energy Plus, and the energy savings per unit area were calculated based on the total floor area. 20 different cities in China were selected for the simulation, including Harbin, Shenyang, Beijing, Shijiazhuang, Jinan, Inner Mongolia, Zhengzhou, Hefei, Shanghai, Lanzhou, Xi'an, Qinghai, Chengdu, Chongqing, Changsha, Guangzhou, Fuzhou, Tibet, Kunming and Sanya, as shown in the **Fig.** S13-S14. (In this study, the KCWO-PNIPAM layer demonstrates two distinct emissivity values, 35 and 47, resulting from its phase transition at varying temperatures. In comparison, the emissivity value of standard glass is 89. The simulation calculations for energy savings in this research utilized the highest value of 47.)

**Table S2** Optical properties for the samples used in actual building energy consumption simulation

|  | Normal glass | KCWO-PNIPAM | PSLC | KCWO-PNIPAM/PSLC | PSLC/KCWO-PNIPAM |
| --- | --- | --- | --- | --- | --- |
| T_lum_ | 88.9 | 20℃-83 | 0V-82.7 | H_on_/E_on_-79 | H_on_/E_on_-79 |
|  |  | 40℃-10.3 | 28V-6.1 | H_off_/E_off_-6.2 | H_off_/E_off_-6.2 |
| T_sol_ | 84.2 | 20℃-57 | 0V-81.7 | H_on_/E_on_-53 | H_on_/E_on_-53 |
|  |  | 40℃-42.8 | 28V-54.3 | H_off_/E_off_-5.7 | H_off_/E_off_-5.7 |
| E_LWIR_-Front | 0.89 | 20℃-25 | 0V-95 | 20℃-35 | 95 |
|  |  | 40℃-53 | 28V-95 | 40℃-47 |  |
| E_LWIR_-Black | 0.89 | 20℃-25 | 0V-95 | 95 | 20℃-35 |
|  |  | 40℃-53 | 28V-95 |  | 40℃-47 |
| R_lum_-Front | 6.5 | 20℃-14.8 | 0V-19.8 | H_on_/E_on_-14.2 | H_on_/E_on_-18.4 |
|  |  | 40℃-34.5 | 28V-34.3 | H_off_/E_off_-34.7 | H_off_/E_off_-34.7 |
| R_lum_-Black | 6.5 | 20℃-14.8 | 0V-19.8 | H_on_/E_on_-18.4 | H_on_/E_on_-14.2 |
|  |  | 40℃-34.5 | 28V-34.3 | H_off_/E_off_-34.7 | H_off_/E_off_-34.7 |
| R_sol_-Front | 6.2 | 20℃-5.43 | 0V-9.8 | H_on_/E_on_-6.23 | H_on_/E_on_-7.24 |
|  |  | 40℃-9.2 | 28V-14.7 | H_off_/E_off_-13.6 | H_off_/E_off_-14.92 |
| R_sol_- Black | 6.2 | 20℃-5.43 | 0V-9.8 | H_on_/E_on_-7.24 | H_on_/E_on_-6.23 |
|  |  | 40℃-9.2 | 28V-14.7 | H_off_/E_off_-14.92 | H_off_/E_off_-13.6 |


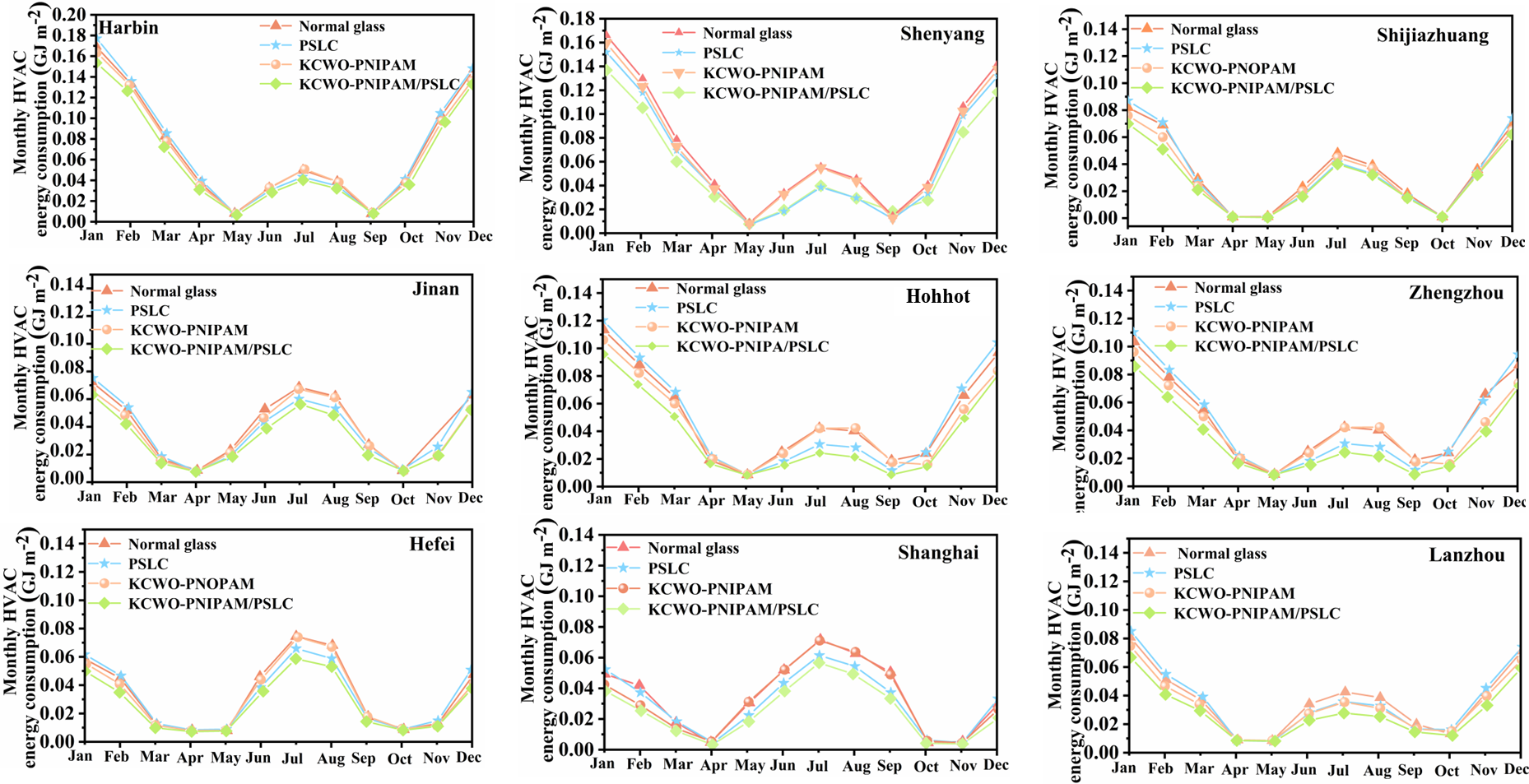


**Fig. S13 (a−i)** Monthly HVAC energy consumption of normal glass, PSLC, KCWO-PNIPAM, and the KPP window in the climate conditions of Harbin, Shenyang, Shijiazhuang, Jinan, Hohhot, Zhengzhou, Hefei, Shanghai and Lanzhou, respectively.


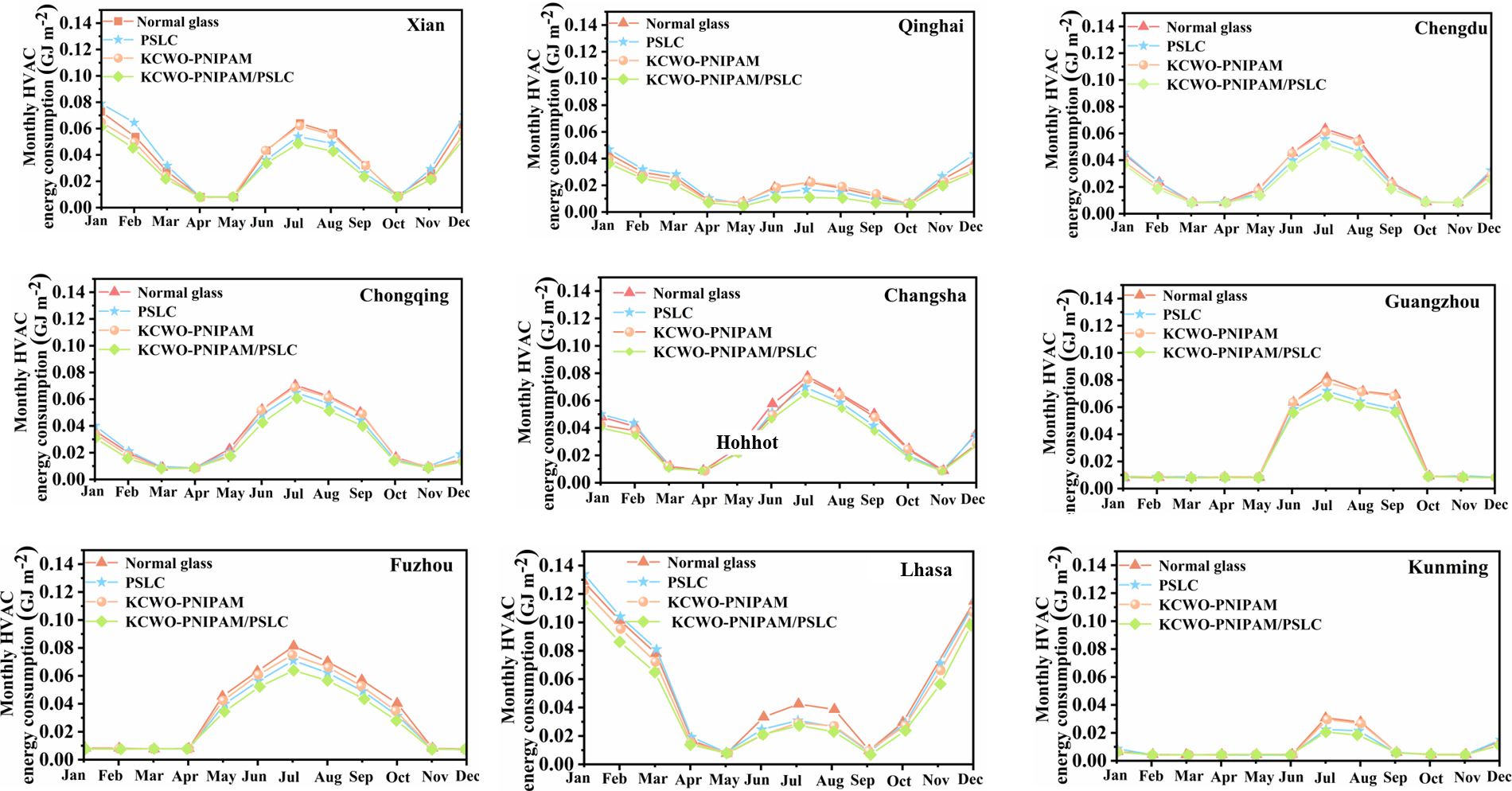


**Fig. S14 (a−i)** Monthly HVAC energy consumption of normal glass, PSLC, KCWO-PNIPAM, and the KPP window in the climate conditions of Xi’an, Qinghai, Chengdu, Chongqing, Changsha, Guangzhou, Fuzhou, Lhasa and Kunming, respectively.





**Fig. S15** Monthly HVAC energy consumption of normal glass, PSLC, KCWO-PNIPAM, and the KPP window in the climate conditions of Sanya.

**S8 Evaluation Criteria for Smart Window Technologies**

To illustrate the advantages of this study, a comprehensive comparison of electrochromic, thermochromic, and liquid crystal-based smart windows is presented from six perspectives: NOM, ASSR, AE, ΔT_lum_, SR, and ES. Compared to reported smart window technologies, this study demonstrates superior performance across multiple aspects, as shown in **Fig.** S15, with specific parameters detailed in Table S3. In summary, this research highlights the integration of optical performance and electromagnetic shielding functionality, which is of significant importance for the comprehensive application of smart windows in multifunctional fields.

**Number of Optical Modes (NOM):** This standard assesses the control modes of smart windows. Devices that support active control, passive control, and can achieve multi-level dynamic control are awarded 3 points, reflecting their adaptability to various environments. Devices with one control mode receive 1 point, while those with two control modes earn 2 points.

**Adjustable solar spectral range (ASSR):** This standard is utilized to assess the effectiveness of smart windows in managing the solar spectrum. A maximum score of three points is awarded for the ability to control the entire spectrum, including ultraviolet, visible, and near-infrared light. This score reflects the window's capability to regulate the solar spectrum comprehensively. A dual-band control capability receives 2 points, while a single-band control capability is awarded 1 point.

**Adjustable Emissivity (AE):** This standard assesses the radiative cooling capability of smart windows. It awards three points for successfully achieving dynamic adjustment of emissivity within the atmospheric window (8-13 μm) and assigns zero points for failing to achieve such dynamic adjustment.

**Visible light transmittance variation (ΔT_lum_):** This standard evaluates the capability of smart windows to regulate visible light. A ΔT_lum_(%) percentage exceeding 75 is deemed excellent, earning three points. A score of two points is awarded for a range of 65 to 75, and one point is given for a range of 55 to 65. Scores below 55 are recorded as zero points.

**Switching Speed (SR):** Given the importance of responsiveness for user convenience, this metric evaluates the time required for a smart window to transit between states. Fast-switching devices, operating at millisecond levels, are regarded highly with a score of 3. Devices with second-level switching speeds are scored at 1, acknowledging their slower response times. Intermediate speeds are awarded a score of 2.

**Electromagnetic shielding effectiveness (ES)：**This standard assesses the shielding effectiveness of smart windows against electromagnetic waves. An electromagnetic shielding (ES) efficiency greater than 30 is awarded 3 points, an ES efficiency between 10 and 30 receives 2 points, and an efficiency of less than 10 is assigned 1 point.


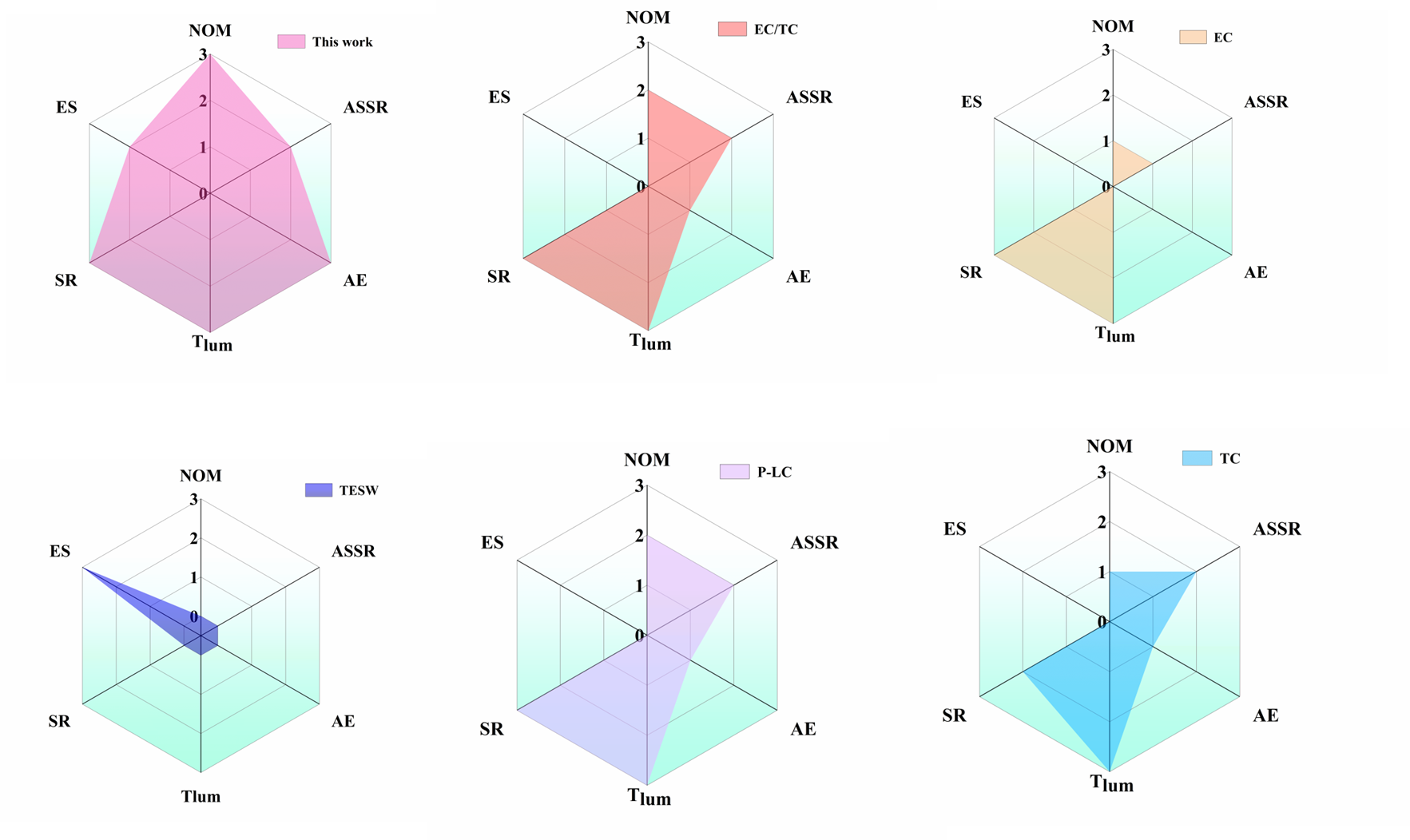


**Fig. S16** Current status analysis of multispectral smart windows

EC, Electrochromic smart window; LC：Liquid crystal material system; P-LC, Polymer-liquid crystal material; TC: thermochromic smart window; TC/EC: electro- and thermochromics smart window;TESW,Transparent electromagnetic shielding window.

**Table S3** Typical smart windows with adjustable optical functions and microwave shielding capabilities. Symbol "-" indicates the terms that have not been concerned or targeted in the study

| type of window | Optical function manipulation | | | | | | Microwave shielding function | | | | Refs. |
| --- | --- | --- | --- | --- | --- | --- | --- | --- | --- | --- | --- |
|  | Number of optical modes | Adjustable solar spectral range | Emissivity at atmospheric windows | ΔT_lum_(%) | Response time  T_on_/to_ff_ | Switching conditions | | Electromagnetic shielding effectiveness  (dB) | | Effective microwave shielding range(GHz) |  |
| PSLC/KCWO-PNIPAM | 3 | Vis/Nir | 0.42-0.95 | 78 | 30ms/2.5ms | 0V-28v  35℃ | | 28.9 | 8.0-12.5 | | This  work |
| EC | 1 | Vis | - | 68 | 0.5 s/0.2 s | -2.5 V- +2.5 V | | Not touched | | | [S15] |
| EC | 1 | Vis | - | 73 | 11.5 s/6 s | -1 V- +1V | | Not touched | | | [S16] |
| EC | 1 | Vis | - | 75 | 6.4 s/7.2 s | -1 V- +1V | | Not touched | | | [S17] |
| EC | 1 | Vis | - | 70 | 12s/6.7s | -1 V- +1 V | | Not touched | | | [S18] |
| EC | 1 | Vis | - | 74.7 | 7.28s/6.4s | -1 V- +1 V | | Not touched | | | [S19] |
| EC | 1 | Vis | - | 73 | 8.26s/5.3s | -1 V- +6 V | | Not touched | | | [S20] |
| EC | 1 | Vis | - | 25 | 22.18s/8.4s | -1 V- +8 V | | Not touched | | | [S21] |
| EC | 1 | Vis | - | 55.9 | 1. 8s/3.4s | -1 V- +5 V | | Not touched | | | [S22] |
| P-LC | 2 | Vis | - | 75 | 40 ms/- | 0 V-50 V | | Not touched | | | [S23] |
| P-LC | 2 | Vis/Nir | - | 76 | 55 ms/- | 0 V-55 V | | Not touched | | | [S24] |
| P-LC | 2 | Vis/Nir | - | 74 | 100 ms/- | 0 V-34.2 V | | Not touched | | | [S25] |
| P-LC | 2 | Vis | - | 76 | 84 ms/- | 0 V-40 V | | Not touched | | | [S26] |
| P-LC | 2 | Vis | - | 77 | 1 ms/0.47 ms | 0 V-30 V | | Not touched | | | [S27] |
| P-LC | 2 | Vis/Nir | - | 79 | 21 ms/6 ms | 0 V-40 V | | Not touched | | | [S28] |

| type of window | Optical function manipulation | | | | | | Microwave shielding function | | | Refs. |
| --- | --- | --- | --- | --- | --- | --- | --- | --- | --- | --- |
|  | Number of optical modes | Adjustable solar spectral range | Adjustable Emissivity | ΔT_lum_(%) | Response time  T_on_/to_ff_ | Switching conditions | Electromagnetic shielding effectiveness  (dB) | Effective microwave shielding range(GHz) | |  |
| LC | 1 | Vis | - | ~70 | - | 0 V-20 V | Not touched | | | [S29] |
| LC | 1 | Vis | - | ~70 | - | 0 V-40 V | Not touched | | | [S30] |
| LC | 1 | Vis | - | ~70 | - | 0 V-20 V | Not touched | | | [S31] |
| LC | 1 | Vis | - | ~70 | 10 ms/100 ms | 0 V-112 V | Not touched | | | [S32] |
| LC | 1 | Vis | - | ~70 | 6.43 ms/44.2 ms | - | Not touched | | | [S33] |
| LC | 1 | Vis | - | ~70 | - | 0 V -12 V -40 V | Not touched | | | [S34] |
| TC | 1 | Vis/Nir | 0.35-0.68 | ~78 | 100s | ~35℃ | Not touched | | | [S35] |
| TC | 1 | Vis/Nir | 0.1-0.8 | ~72 | 100s | ~35℃ | Not touched | | | [S36] |
| TC | 1 | Vis/Nir | - | ~75 | 100s | ~35℃ | Not touched | | | [S37] |
| TC/EC | 2 | Vis/Nir | - | ~78 | 12s/6.7s | -1 V- +1 V | Not touched | | | [S38] |
| TC/EC | 2 | Vis/Nir | - | ~78 | 22.18s/8.4s | -1 V- +5 V | Not touched | | | [S39] |
| TESW | 0 | - | Not touched | | | | 32 | | 8.2-12.4 | [S40] |
| TESW | 0 | - | Not touched | | | | 25 | | 8.2-12.5 | [S41] |
| TESW | 0 | - | Not touched | | | | 37.5 | | 8.2-12.4 | [S42] |

ΔT_lum_, the visible light transmittance variation; EC, Electrochromic smart window; LC：Liquid crystal material system; P-LC, Polymer-liquid crystal material; TC: thermochromic smart window; TC/EC: electro- and thermochromics smart window;TESW,Transparent electromagnetic shielding window.

**References**

1. S. Zhong, L. Yi, J. Zhang, T. Xu, L. Xu, X. Zhang, T. Zuo, Y. Cai, Self-cleaning and spectrally selective coating on cotton fabric for passive daytime radiative cooling, Chem. Eng. J. **407**, 127104 (2021). <https://doi.org/10.1016/j.cej.2020.127104>
2. J. Mandal, Y. Fu, A.C. Overvig, M. Jia, K. Sun et al., Hierarchically porous polymer coatings for highly efficient passive daytime radiative cooling. Science **362**(6412), 315–319 (2018). <https://doi.org/10.1126/science.aat9513>
3. C. Ma, Z. Zhang, Y. Yang, P. Wang, M. Yu et al., A smart window with passive radiative cooling and switchable near-infrared light transmittance *via* molecular engineering. ACS Appl. Mater. Interfaces **16**(19), 25343–25352 (2024). <https://doi.org/10.1021/acsami.4c02819>
4. Z. Zhang, Y. Yang, C. Ma, M. Yu, J. Xu et al., Enhanced electro-optical and heat regulation of intelligent dimming films using the photovoltaic effect of p–n heterostructures. Adv. Funct. Mater. **34**(45), 2406858 (2024). <https://doi.org/10.1002/adfm.202406858>
5. S. Wang, Y. Zhou, T. Jiang, R. Yang, G. Tan et al., Thermochromic smart windows with highly regulated radiative cooling and solar transmission. Nano Energy **89**, 106440 (2021). <https://doi.org/10.1016/j.nanoen.2021.106440>
6. ANSI/ASHRAE/IES, Energy standard for building except low-rise residential buildings, 2019.
7. M. Deru, K. Field, D. Studer, K. Benne, B. Griffith, P. Torcellini, B. Liu, M. Halverson, D. Winiarski, M. Rosenberg, US Department of Energy commercial reference building models of the national building stock, 2011.
8. J.-H. Choi, V. Loftness, A. Aziz, Post-occupancy evaluation of 20 office buildings as basis for future IEQ standards and guidelines. Energy Build. **46**, 167–175 (2012). <https://doi.org/10.1016/j.enbuild.2011.08.009>
9. X. Chen, Y. Gu, J. Liang, M. Bai, S. Wang et al., Enhanced microwave shielding effectiveness and suppressed reflection of chopped carbon fiber felt by electrostatic flocking of carbon fiber. Compos. Part A Appl. Sci. Manuf. **139**, 106099 (2020). <https://doi.org/10.1016/j.compositesa.2020.106099>
10. K. Koo, E.H. Won, S.H. Lee, Y.M. Park, J.Y. Yu, Electromagnetic Interference (EMI) Shielding Effectiveness (SE) of PET Fabrics Plated with Stainless Steel Thin Layer by Sputtering. Text. Sci. Eng. **43**, 101–106 (2006). <https://doi.org/10.1016/S0379-6779(01)00332-0>
11. S. Sharma, J. Lee, T.T. Dang, S.H. Hur, W.M. Choi et al., Ultrathin freestanding PDA-Doped rGO/MWCNT composite paper for electromagnetic interference shielding applications. Chem. Eng. J. **430**, 132808 (2022). <https://doi.org/10.1016/j.cej.2021.132808>
12. B.R. Tao, J. Li, F.J. Miao, Y. Zang, Carbon cloth loaded nico_2_o_4_ nano-arrays to construct Co-MOF@GO nanocubes: a high-performance electrochemical sensor for non-enzymatic glucose. IEEE Sens. J. **22**, 13898–13907 (2022). <https://doi.org/10.1109/JSEN.2022.3170026>
13. K. Wang, C. Chen, Q. Zheng, J. Xiong, H. Liu et al., Multifunctional recycled carbon fiber-Ti3C2Tx MXene paper with superior electromagnetic interference shielding and photo/electro-thermal conversion performances. Carbon **197**, 87–97 (2022). <https://doi.org/10.1016/j.carbon.2022.06.026>
14. J. Cheng, C. Li, Y. Xiong, H. Zhang, H. Raza et al., Recent advances in design strategies and multifunctionality of flexible electromagnetic interference shielding materials. Nano-Micro Lett. **14**(1), 80 (2022). <https://doi.org/10.1007/s40820-022-00823-7>
15. B. Deng, Y. Zhu, X. Wang, J. Zhu, M. Liu et al., An ultrafast, energy-efficient electrochromic and thermochromic device for smart windows. Adv. Mater. **35**(35), 2302685 (2023). <https://doi.org/10.1002/adma.202302685>
16. Q. Zhao, Y. Fang, K. Qiao, W. Wei, Y. Yao et al., Printing of WO_3_/ITO nanocomposite electrochromic smart windows. Sol. Energy Mater. Sol. Cells **194**, 95–102 (2019). <https://doi.org/10.1016/j.solmat.2019.02.002>
17. S. Zhang, Y. Peng, J. Zhao, Z. Fan, B. Ding et al., amorphous and porous tungsten oxide films for fast‐switching dual‐band electrochromic smart windows. Adv. Optical Mater. **11,** 2202115 (2023). <https://doi.org/10.1002/adom.202202115>
18. M.T. Zahoor, M. Ahmad, K. Maaz, S. Karim, K. Waheed et al., Tungsten oxide multifunctional nanostructures: Enhanced environmental and sensing applications. Mater. Chem. Phys. **221**, 250–257 (2019). <https://doi.org/10.1016/j.matchemphys.2018.09.034>
19. Y. Liu, N. Jiang, Y. Liu, D. Cui, C.-F. Yu et al., Effect of laser power density on the electrochromic properties of WO_3_ films obtained by pulsed laser deposition. Ceram. Int. **47**(16), 22416–22423 (2021). <https://doi.org/10.1016/j.ceramint.2021.04.251>
20. T. Ma, B. Li, S. Tian, J. Qian, L. Zhou et al., Reversible photochromic W^18^O_49_: Mechanism revealing and performance improvement for smart windows. Chem. Eng. J. **468**, 143587 (2023). <https://doi.org/10.1016/j.cej.2023.143587>
21. W. Cheng, J. He, K.E. Dettelbach, N.J.J. Johnson, R.S. Sherbo et al., Photodeposited Amorphous Oxide Films for Electrochromic Windows. Chem **4,** 821-832 (2018). <https://doi.org/10.1016/j.chempr.2017.12.030>
22. L. Shen, L. Du, S. Tan, Z. Zang, C. Zhao et al., Flexible electrochromic supercapacitor hybrid electrodes based on tungsten oxide films and silver nanowires. Chem. Commun. **52**(37), 6296–6299 (2016). <https://doi.org/10.1039/C6CC01139J>
23. A. Katariya-Jain, R.R. Deshmukh, Effects of dye doping on electro-optical, thermo-electro-optical and dielectric properties of polymer dispersed liquid crystal films. J. Phys. Chem. Solids **160**, 110363 (2022). <https://doi.org/10.1016/j.jpcs.2021.110363>
24. X. Liang, C. Guo, M. Chen, S. Guo, L. Zhang et al., A roll-to-roll process for multi-responsive soft-matter composite films containing CsxWO_3_ nanorods for energy-efficient smart window applications. Nanoscale Horiz. **2**, 319-325 (2017). <https://doi.org/10.1039/C7NH00105C>
25. Liang, X. et al. Dual-Band Modulation of Visible and Near-Infrared Light Transmittance in an All-Solution-Processed Hybrid Micro-Nano Composite Film. **9**, 40810-40819 (2017). <https://doi.org/10.1021/acsami.7b11582>
26. M. Kim, K.J. Park, S. Seok, J.M. Ok, H.-T. Jung et al., Fabrication of microcapsules for dye-doped polymer-dispersed liquid crystal-based smart windows. ACS Appl. Mater. Interfaces **7**(32), 17904–17909 (2015). <https://doi.org/10.1021/acsami.5b04496>
27. X. Hu, X. Zhang, W. Yang, X.-F. Jiang, X. Jiang et al., Stable and scalable smart window based on polymer stabilized liquid crystals. J. Appl. Polym. Sci. **137**(30), 48917 (2020). <https://doi.org/10.1002/app.48917>
28. Z. Zhang, R. Zhang, L. Xu, J. Li, L. Yang et al., Visible and infrared optical modulation of PSLC smart films doped with ATO nanoparticles. Dalton Trans. **50**(29), 10033–10040 (2021). <https://doi.org/10.1039/d1dt01575c>
29. Y. Zhan, A.P.H.J. Schenning, D.J. Broer, G. Zhou, D. Liu, Light-driven electrohydrodynamic instabilities in liquid crystals. Adv. Funct. Mater. **28**(21), 1707436 (2018). <https://doi.org/10.1002/adfm.201707436>
30. J.-W. Huh, J.-H. Kim, S.-W. Oh, S.-M. Ji, T.-H. Yoon, Ion-doped liquid-crystal cell with low opaque-state specular transmittance based on electro-hydrodynamic effect. Dyes Pigm. **150**, 16–20 (2018). <https://doi.org/10.1016/j.dyepig.2017.11.001>
31. J.-W. Huh, J.-H. Seo, S.-W. Oh, S.-H. Kim, T.-H. Yoon et al. Tristate switching of a liquid-crystal cell among initial transparent, haze-free dark, and high-haze dark states. J. Mol. Liq. **281**, 81-85 (2019). <https://doi.org/10.1016/j.molliq.2019.02.082>
32. C.-W. Chen, A.N. Brigeman, T.-J. Ho, I.C. Khoo, Normally transparent smart window based on electrically induced instability in dielectrically negative cholesteric liquid crystal. Opt. Mater. Express **8**(3), 691 (2018). <https://doi.org/10.1364/ome.8.000691>
33. C.-H. Han, S.-W. Oh, A high-haze liquid crystal grating device with asymmetric anchoring energies. Displays **81**, 102581 (2024). <https://doi.org/10.1016/j.displa.2023.102581>
34. J.-W. Huh, S.-W. Oh, J.-H. Seo, H.-J. Sohn, T.-H. Yoon, Filtering of yellow light in a liquid-crystal light shutter for higher color contrast and reduced glare. J. Mol. Liq. **327**, 114846 (2021). <https://doi.org/10.1016/j.molliq.2020.114846>
35. R. Zhang, R. Li, P. Xu, W. Zhong, Y. Zhang et al., Thermochromic smart window utilizing passive radiative cooling for self-adaptive thermoregulation. Chem. Engin. J. **471**, 144527, (**2023).** <https://doi.org/10.1016/j.cej.2023.144527>
36. S. Wang, Y. Zhou, T. Jiang, R. Yang, G. Tan et al., Thermochromic smart windows with highly regulated radiative cooling and solar transmission. Nano Energy **89**, 106440 (2021). <https://doi.org/10.1016/j.nanoen.2021.106440>
37. M. Wang, S. Liang, S. Zhao, W. Gao, Z. Li, Facile preparation of a low-cost liquid interlayer material with intelligent UV−NIR-shielding function for smart windows. ACS Appl. Mater. Interfaces **15,** 48673–48682 (2023). https://doi.org/10.1021/acsami.3c10909
38. L. Zhang, Y. Du, F. Xia, Y. Gao, Two birds with one stone: a novel thermochromic cellulose hydrogel as electrolyte for fabricating electric-/ thermal-dual-responsive smart windows. Chem. Eng. J. **455**, 140849 (2023). <https://doi.org/10.1016/j.cej.2022.140849>
39. S.Z. Sheng, J.L. Wang, B. Zhao et al., Nanowire-based smart windows combining electro- and thermochromics for dynamic regulation of solar radiation]. Nat. Commun. **14**(1), 3231 (2023). https://doi.org/10.1038/s41467-023-38353-4
40. B. Zhou, Flexible MXene/silver nanowires-based transparent conductive film with electromagnetic interference shielding and electro-photo-thermal performances. ACS Appl. Mater. Interfaces **12**, 40859–40869 (2020). <https://pubs.acs.org/doi/10.1021/acsami.0c09020>
41. X. Liang, T. Zhao, P. Zhu, Y. Hu, R. Sun et al., Room-temperature nanowelding of a silver nanowire network triggered by hydrogen chloride vapor for flexible transparent conductive films. ACS Appl. Mater. Interfaces **46**, 40857-40867 (2017). <https://doi.org/10.1021/acsami.7b13048>
42. C.-M. Cheng, Y.-H. Chen, S.-Y. Lin, S.-D. Chao, S.-F. Tsai, Study on shielding effectiveness of high transmittance coating film glasses against electromagnetic pulse. Technologies **11**(6), 175 (2023). <https://doi.org/10.3390/technologies11060175>
